# Calcium enhances hydrolytic and transfucosylation activities in α-L-fucosidase from *Thermotoga maritima* through catalytic loop stabilization: an MD simulation study

**DOI:** 10.1007/s00253-026-13881-3

**Published:** 2026-05-23

**Authors:** Catalina Torres-Ochoa, Carlos Jiménez-Pérez, Salvador R. Tello-Solís, Sergio Alatorre-Santamaría, Francisco Guzmán-Rodríguez, Lorena Gómez-Ruiz, Alma Cruz-Guerrero

**Affiliations:** 1 Departamento de Biotecnología, Universidad Autónoma Metropolitana-Iztapalapa, Av. San Rafael Atlixco 186, Col. Vicentina, Mexico, 09340 Mexico; 2 Departamento de Química, Universidad Autónoma Metropolitana-Iztapalapa, Av. San Rafael Atlixco 186, Col. Vicentina, Mexico, 09340 Mexico; 3 Departamento de Alimentos y Biotecnología, Facultad de Química, UNAM. Circuito Exterior, Ciudad Universitaria, Mexico, 04510 Mexico

**Keywords:** α-L-Fucosidase, Transfucosylation, Fucosylated oligosaccharides, Glycoside hydrolases, Molecular dynamics simulation, Calcium ions, Enzyme regulation, Catalytic loop dynamics

## Abstract

**Abstract:**

α-L-Fucosidases are versatile glycoside hydrolases capable of catalyzing both hydrolysis and transfucosylation reactions, making them valuable biocatalysts for the synthesis of fucosylated oligosaccharides (FucOS). However, transfucosylation efficiency is often limited by competing hydrolysis, and the factors governing this balance remain incompletely understood. In this study, we investigated the effect of divalent cations on the catalytic behavior of the α-L-fucosidase from *Thermotoga maritima* (FUC-*Tm*) by combining enzymatic assays with molecular dynamics (MD) simulations and binding energy analyses. Among the tested ions, Ca^2+^ produced the most pronounced effect, increasing both hydrolytic and transfucosylation activities, with the highest transfucosylation efficiency observed at intermediate CaCl_2_ concentrations. MD simulations indicated that Ca^2+^ does not induce major global conformational changes but is associated with reduced flexibility in loop regions surrounding the catalytic pocket, particularly Loop-B, and with improved positioning of catalytic residues. These effects are consistent with enhanced substrate interactions, as supported by binding energy analyses. However, the increase in transfucosylation efficiency coincided with a decrease in water activity, suggesting that the observed behavior arises from a combination of structural and physicochemical factors. Therefore, Ca^2+^-mediated modulation of FUC-*Tm* activity is best interpreted as a multifactorial process involving changes in local protein dynamics and in the reaction environment. These findings provide insight into ion-dependent modulation of glycoside hydrolases and highlight the importance of integrating structural dynamics and solvent effects to improve enzymatic synthesis of FucOS.

**Key points:**

- *Calcium enhances both hydrolytic and transfucosylation activities of α-L-fucosidase from Thermotoga maritima under specific conditions.*
- *Molecular dynamics simulations indicate that Ca*^*2+*^* is associated with reduced flexibility in loop regions adjacent to the catalytic site, contributing to the maintenance of catalytic pocket geometry.*
- *The modulation of catalytic behavior is influenced by a combination of structural effects and physicochemical factors, including changes in water activity and ionic strength.*

**Supplementary Information:**

The online version contains supplementary material available at 10.1007/s00253-026-13881-3.

## Introduction

Fucosylated oligosaccharides (FucOS) are structurally diverse carbohydrates that play important biological roles in cell–cell recognition, host–microbe interactions, and modulation of the immune system (Rudloff and Kunz 2012; Zhao et al. 2017; Zehra et al. 2018). These molecules have attracted considerable attention due to their presence in human milk oligosaccharides and their potential applications in functional foods, nutraceuticals, and therapeutic formulations (Krupinskaitė et al. 2024). Consequently, the development of efficient strategies for the synthesis of FucOS has become an important objective in glycobiotechnology. Enzymatic synthesis using glycoside hydrolases has emerged as an attractive alternative to traditional chemical synthesis because of its high regioselectivity, stereoselectivity, and environmentally friendly reaction conditions (Zeuner and Meyer 2020; Liu et al. 2022).

Among the enzymes involved in carbohydrate metabolism, α-L-fucosidases (EC 3.2.1.51) catalyze the hydrolysis of terminal α-L-fucosyl residues from glycoconjugates and oligosaccharides. In addition to hydrolysis, several α-L-fucosidases can catalyze transglycosylation reactions, transferring a fucosyl residue from a donor substrate to an acceptor molecule to form new glycosidic linkages. This catalytic promiscuity has made these enzymes valuable tools for the enzymatic synthesis of FucOS. However, the efficiency of transfucosylation reactions is often limited by the competing hydrolytic activity of the enzyme, which frequently reduces product yields and represents a major challenge for the practical application of these biocatalysts (Bridiau et al. 2010; Vera et al. 2017; Robles-Arias et al. 2021).

Various strategies have been explored to enhance transglycosylation reactions catalyzed by glycoside hydrolases. These include protein engineering approaches, optimization of donor and acceptor concentrations, and modulation of reaction conditions such as temperature, solvent composition, and water activity (*a*_w_). Recent advances in glycobiotechnology have also focused on improving the efficiency of transfucosylation reactions through enzyme engineering and optimization of reaction conditions. Several studies have demonstrated that modifications in regions surrounding the catalytic pocket of glycoside hydrolases can significantly enhance transglycosylation yields while reducing competing hydrolysis reactions (Zeuner et al. 2019; Coines et al. 2022). In this context, recent reviews highlight the growing interest in enzymatic strategies for the synthesis of FucOS and the development of improved α-L-fucosidase-based biocatalysts (Zeuner and Meyer 2020; Liu et al. 2022; Zheng et al. 2022; Moya-Gonzálvez et al. 2024).

Recent studies have also suggested that divalent metal ions may influence the structural dynamics and catalytic behavior of glycoside hydrolases. In several glycoside hydrolase (GH) families, Ca^2+^ and other metal ions have been reported to stabilize flexible structural elements or regions adjacent to catalytic pockets, thereby influencing substrate binding or the geometry of catalytically competent conformations. For example, presence of Ca^2+^ has been associated with stabilization of loop regions within the catalytic domain of a GH13 dextran glucosidase (Kobayashi et al. 2011), while structural studies of a GH115 α-glucuronidase revealed a Ca^2+^ ion coordinated by loop elements that participate directly in substrate interactions (Wilkens et al. 2022). In other systems, such as GH43 arabinanases, divalent cations have been proposed to modulate the catalytic microenvironment through electrostatic effects on residues involved in proton transfer (de Sanctis et al. 2010). Collectively, these observations suggest that metal ions may influence catalytic efficiency in glycoside hydrolases by modulating local structural flexibility or the architecture of the active-site pocket.

Despite these advances, the molecular basis by which divalent cations influence the catalytic behavior of α-L-fucosidases remains poorly understood. In particular, it is unclear whether these effects arise from specific structural interactions with the enzyme or from indirect physicochemical factors such as ionic strength or water activity. Addressing this question is essential for improving enzymatic strategies for FucOS synthesis. The α-L-fucosidase from *Thermotoga maritima* (FUC-*Tm*) represents a suitable model system due to its thermostability and catalytic versatility. This enzyme belongs to glycoside hydrolase family 29 (GH29), a group of retaining enzymes that catalyze the cleavage of α-L-fucosidic linkages through a double-displacement mechanism involving a nucleophile and an acid/base catalyst. Structurally, FUC-*Tm* forms a hexameric assembly, with each subunit containing an independent catalytic site. It exhibits broad substrate specificity, being capable of catalyzing both hydrolytic and transfucosylation reactions, in which fucosyl residues are transferred to acceptor molecules such as lactose to form fucosylated oligosaccharides. Consistent with its thermophilic origin, FUC-*Tm* displays optimal activity at elevated temperatures and acidic pH (Guzmán-Rodríguez et al. 2018; Robles-Arias et al. 2021; Pavón-Chimal et al. 2022). In this study, we investigated the influence of divalent cations on the catalytic behavior of FUC-*Tm* by combining enzymatic assays with molecular dynamics (MD) simulations and binding energy analyses. Rather than assuming a single dominant mechanism, we hypothesize that Ca^2+^ modulates catalytic behavior through a combination of structural effects on local protein dynamics and physicochemical factors, including changes in water activity and ionic strength. Therefore, our aim is to provide an integrated mechanistic framework that accounts for both structural and environmental contributions to enzyme modulation.

## Materials and methods

### Chemicals and enzyme source

*p*-Nitrophenyl α-L-fucopyranoside (*p*NP-Fuc), 4-nitrophenol (*p*NP), D-lactose, L-fucose, and 2′-fucosyllactose were obtained from commercial sources. Calcium chloride (CaCl_2_), magnesium chloride (MgCl_2_), and manganese chloride (MnCl_2_) were of analytical grade.

The α-L-fucosidase from *Thermotoga maritima* (FUC-*Tm*) was purchased from Megazyme (Bray, Ireland) with an activity of 5 U/mL (2.6 U/mg). The enzyme stock solution, supplied in 3.2 M ammonium sulfate, was diluted 1:1000 prior to use, resulting in a final ammonium sulfate concentration of approximately 3.2 mM, which is considered negligible relative to the ionic strength conditions evaluated in this study. The purity of the enzyme preparation has been previously assessed by SDS-PAGE, showing a single band corresponding to the expected molecular weight of α-L-fucosidase (Guzmán-Rodríguez et al. 2018).

### Effect of divalent ions on hydrolytic activity

The hydrolytic activity of FUC-*Tm* was determined using a reaction mixture containing 3.5 mM *p*NP-Fuc as the substrate. Enzyme activity was measured with 0.00325 U/mL (3.12 × 10^–8^ M) of FUC-*Tm* in 1 mM sodium acetate buffer (pH 5.0). All experiments were conducted at 60 °C, corresponding to the optimal temperature range reported for FUC-*Tm*, in order to ensure maximal catalytic activity and to allow consistent evaluation of the effect of divalent cations under controlled conditions. Reactions were incubated for 20 min at 60 °C in the presence of 0.25, 0.5, 0.75, 1, 1.25, and 1.5 M of CaCl_2_, MgCl_2_, or MnCl_2_. A control reaction without ions was also performed. Under these conditions, CaCl_2_ was present in large excess relative to the enzyme (millimolar vs. nanomolar concentrations), resulting in a high Ca^2+^ enzyme molar ratio, which does not correspond to a defined binding stoichiometry.

The hydrolytic activity was determined from the release of *p*NP from *p*NP-Fuc in the absence of any added acceptor substrate (e.g., lactose). Under these conditions, transfucosylation reactions are not expected to occur to a significant extent due to the lack of suitable acceptor molecules; therefore, the measured rate predominantly reflects hydrolytic activity.

The amount of *p*NP released was determined by visible spectrophotometry at 410 nm using a UV-1601 spectrophotometer (Shimadzu, Kyoto, Japan) coupled to a TCC temperature controller (Shimadzu, Japan), taking readings every minute. Quantification was carried out by interpolation on a standard *p*NP curve (0.72 to 3.6 mM). One unit of fucosidase was defined as the amount of enzyme required to hydrolyze 1 μmol of *p*NP-Fuc per minute at pH 5.0 and 60 °C. Hydrolytic activity was expressed as U/mL. All experiments were performed in triplicate.

### Effect of calcium on transfucosylation activity

The transfucosylation activity of FUC-*Tm* was evaluated using a reaction mixture containing 3.5 mM *p*NP-Fuc as the donor substrate, 146 mM D-lactose as the acceptor substrate, and 0.0065 U/mL (6.24 × 10^–8^ M) of FUC-*Tm* in 1 mM sodium acetate buffer (pH 5.0). Reactions were incubated at 60 °C for 3 h in a Combi-SV12 hybridization incubator with orbital shaking (FINEPCR, South Korea). Samples were taken every 30 min until 3 h and stopped with 0.3 M NaOH. Activity was measured spectrophotometrically as described previously, both in the absence and presence of 0.25, 0.5, 1, and 1.5 M CaCl_2_. Additionally, water activity (*a*_w_) of the reaction mixtures was measured using an AQUALAB PRE instrument (Decagon Devices, USA) according to the manufacturer’s instructions. Measurements were performed at room temperature. The concentration of synthesized FucOS was determined as described in the “Oligosaccharide quantification” section, where transfucosylation activity was expressed as mM of synthesized FucOS per minute at pH 5.0 and 60 °C.

### Oligosaccharide quantification

The FucOS produced during transfucosylation reactions were quantified by high-performance liquid chromatography (HPLC) using a Lab Alliance Series II pump (Scientific Systems, Pennsylvania, USA) and an HC-75 column (H⁺, 300 × 7.80 mm; Hamilton, Nevada, USA) maintained at 75 °C. Deionized water (Milli-Q®) was used as the mobile phase at a flow rate of 0.3 mL/min. Detection was performed using a light scattering detector (SOFTA 300S ELSD; Teledyne ISCO, Nebraska, USA) with a nebulizer temperature of 10 °C and an evaporator temperature of 45 °C, coupled to a NiGen MICRO nitrogen generator (Claind, Italy) at 62.5 psi. FucOS concentrations were calculated using Clarity software version 5.0.3.180 (DataApex, Czech Republic) by interpolation of peak areas against a 2′ fucosyllactose standard curve.

### In silico study of calcium interaction with the enzyme

#### Molecular dynamics simulations

The crystallographic structure of FUC-*Tm* in its apo form (PDB ID: 2ZWY; resolution: 2.75 Å) (Wu et al. 2010) was retrieved from the Protein Data Bank (RCSB-PDB) (Berman et al. 2000). Only chain A from the hexameric assembly was retained for simulations, and all nonprotein molecules were removed using UCSF Chimera (Version 1.15) (Meng et al. 2006).

Although the crystal structure of FUC-*Tm* corresponds to a hexameric assembly, each subunit contains a structurally self-contained and solvent-exposed active site. Structural inspection suggests that the catalytic residues and nearby flexible regions are not directly involved in inter-subunit interfaces. However, since FUC-*Tm* functions as a hexamer, potential effects of inter-subunit interactions on loop dynamics cannot be fully excluded. Therefore, the present simulations using a monomeric model are intended to provide insight into local structural behavior rather than a complete representation of the native oligomeric system. This approach is consistent with previous in silico studies on GH29 α-L-fucosidases, where monomeric models have been used to investigate catalytic mechanisms and enzyme–substrate interactions (Sulzenbacher et al. 2004; Klontz et al. 2020; Pérez-Escalante et al. 2021; Robles-Arias et al. 2025).

Two systems were constructed: one in the absence of Ca^2+^ (FUC-*Tm*) and another in the presence of calcium (FUC-*Tm*–Ca^2+^), using CaCl_2_ at the optimal concentration previously determined for transfucosylation activity in vitro.

Structural preparation for MD simulations was performed using the CHARMM-GUI server (Lee et al. 2016), with the Solution Builder module. Protonation states of titratable residues were assigned according to their predicted pKa values, corresponding to pH 5.0. Each system was solvated in a cubic TIP3P water box under periodic boundary conditions, maintaining a minimum distance of 10 Å between the protein surface and the box edges. Systems were neutralized by adding Na⁺ and acetate (C_2_H₃O_2_⁻) ions consistent with the experimental buffer conditions. For the FUC-*Tm*–Ca^2+^ system, Ca^2+^ and corresponding Cl^−^ counterions were additionally included.

MD simulations were performed using GROMACS 2023.2 (Abraham et al. 2015), with the CHARMM36 force field. Energy minimization was conducted using the steepest descent algorithm until convergence (maximum force ≤ 10 kJ/mol/nm) or up to 50,000 steps. Two equilibration phases were then carried out: a 100 ps NVT ensemble followed by a 100 ps NPT ensemble at 333.15 K and 1 atm. Temperature and pressure were controlled using the V-rescale thermostat and the Parrinello–Rahman barostat, respectively. Production simulations were subsequently performed for 500 ns in triplicate for each system.

Prior to analysis, trajectories were centered on the protein and corrected for periodic boundary conditions. Structural stability and conformational dynamics were evaluated through root mean square deviation (RMSD) of backbone heavy atoms, root mean square fluctuation (RMSF) per residue, and radius of gyration (Rg). Changes in catalytic pocket volume over time were quantified using Caver Analyst 2.0.

### Ligand optimization and docking

The three-dimensional structure of *p*NP-Fuc (PubChem CID: 82473) (Kim et al. 2023) was geometry-optimized using density functional theory (DFT) with the Perdew exchange–correlation functional (Perdew et al. 1996) and the 6–311 + + G** base set, as implemented in Gaussian 09 Rev A.02 (Frisch et al. 2016).

Molecular docking of *p*NP-Fuc into the apo FUC-*Tm* structure (PDB ID: 2ZWY) was carried out using AutoDockTools (Morris et al. 2009). The search grid (80 × 80 × 80 Å) was centered on the catalytic site based on the coordinates of fucose in PDB ID: 1ODU. Docking calculations employed the Lamarckian Genetic Algorithm with 200 independent runs, an initial population size of 150 individuals, a maximum of 27,000 generations, and 2.5 × 10⁷ energy evaluations. The binding pose with the lowest predicted free energy (Δ*G*) was selected for further analysis. Protein–ligand interactions were visualized using VMD v1.9.3 (Humphrey et al. 1996), PyMOL v2.4.0 (Schrödinger LLC 2015), and BIOVIA Discovery Studio Visualizer v20.1 5 (BIOVIA, Dassault Systèmes 2021).

### Molecular dynamics simulation of the FUC-*Tm*–*p*NP-Fuc complex

The select FUC-*Tm*–*p*NP-Fuc complex was used to generate two additional systems (with and without calcium), which were prepared and equilibrated following the same protocol described above. In this case, production runs were performed for 100 ns in triplicate.

Binding free energies (Δ*G*_u_) were estimated using the molecular mechanics/Poisson–Boltzmann surface area (MM/PBSA) approach implemented in the MMPBSA.py tool (Valdés-Tresanco et al. 2021). Trajectory processing and visualization were performed using VMD v1.9.3, while graphical representations were generated using Grace–WYSIWYG (Grace Development Team n.d.), PyMOL v2.4.0, and BIOVIA Discovery Studio Visualizer v20.1.0.1929528.

### Statistical analysis

All assays were performed in triplicate for each treatment. Results are expressed as mean ± standard deviation. Statistical analysis was conducted using IBM SPSS Statistics for Windows (IBM, NY, USA). Differences between groups were evaluated using Tukey’s test, and a *p*-value < 0.05 was considered statistically significant.

## Results

### Effect of divalent ions on hydrolytic activity

**Fig. 1:**
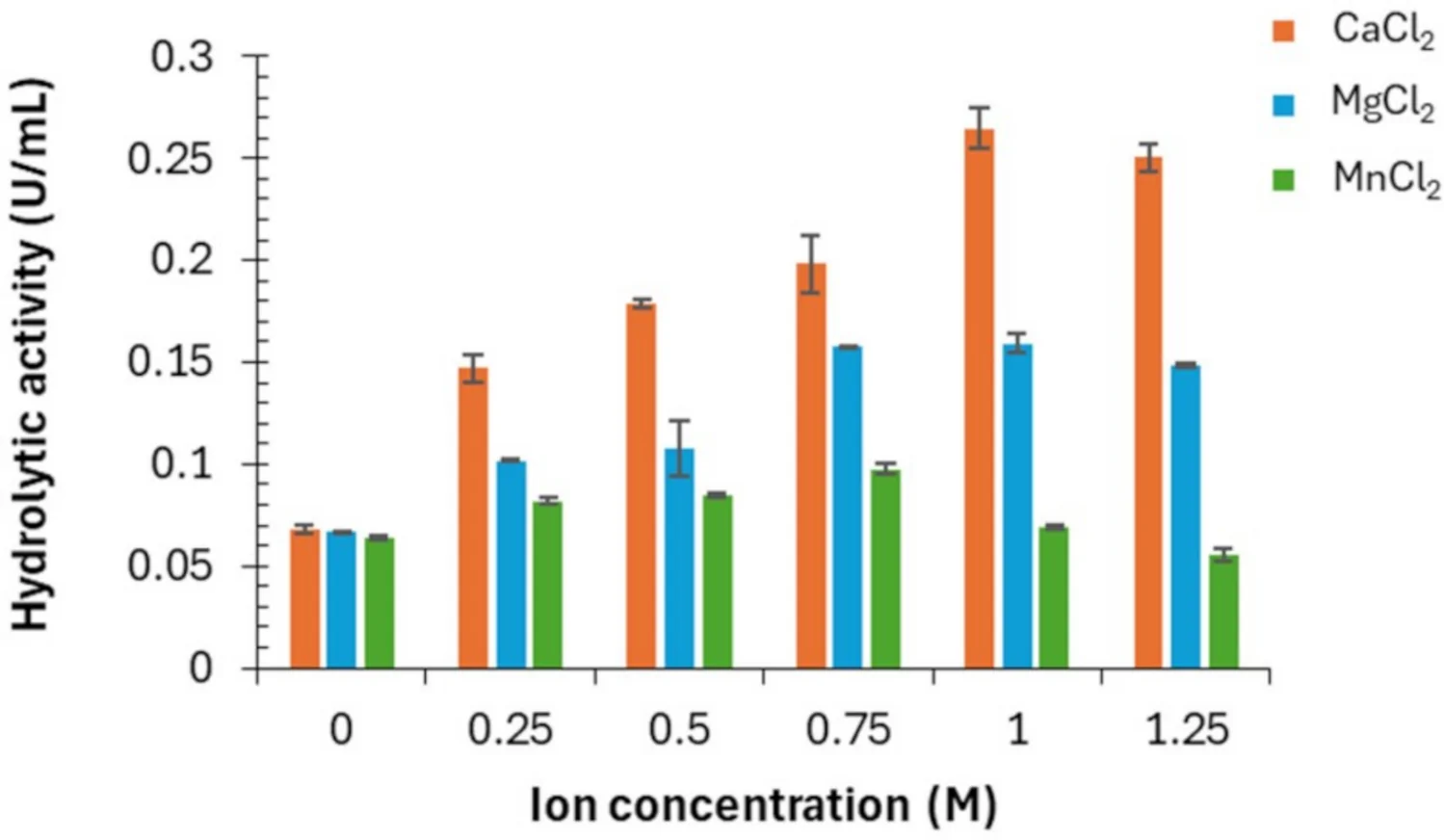
Effect of divalent ions on the hydrolytic activity of FUC-*Tm*. Hydrolytic activity was measured in the presence of increasing concentrations of CaCl_2_, MgCl_2_, and MnCl_2_ using *p*NP-Fuc as substrate in 1 mM acetate buffer (pH 5.0) at 60 °C. Data are presented as mean ± standard deviation (*n* = 3)

The hydrolytic activity of FUC-*Tm* was evaluated in the presence of increasing concentrations of CaCl_2_, MgCl_2_, and MnCl_2_. As shown in Fig. 1, enzyme activity increased with rising salt concentration for all tested ions, although the magnitude of activation differed among them.

In the case of CaCl_2_, the highest activity was observed at concentrations between 1 and 1.25 M, reaching an average value of 0.265 ± 0.01 U/mL, corresponding to approximately 280% increase relative to the control reaction. Similarly, MgCl_2_ enhanced enzyme activity, with a maximum increase of 138% at 0.75 M. The activation range for Mg^2+^ was broader (0.75–1.25 M), although the overall magnitude of activation was lower than that observed with CaCl_2_.

In contrast, MnCl_2_ produced a more moderate effect, with a maximum activation of 53% at 0.75 M, after which enzyme activity decreased at higher concentrations.

### Effect of calcium on transfucosylation activity

Because Ca^2+^ produced the strongest activation of hydrolytic activity, its effect on the transfucosylation reaction responsible for FucOS synthesis was further evaluated. Reactions were performed using CaCl_2_ concentrations of 0.25, 0.5, 1.0, and 1.5 M, and a control reaction without Ca^2+^ was included for comparison.

**Fig. 2:**
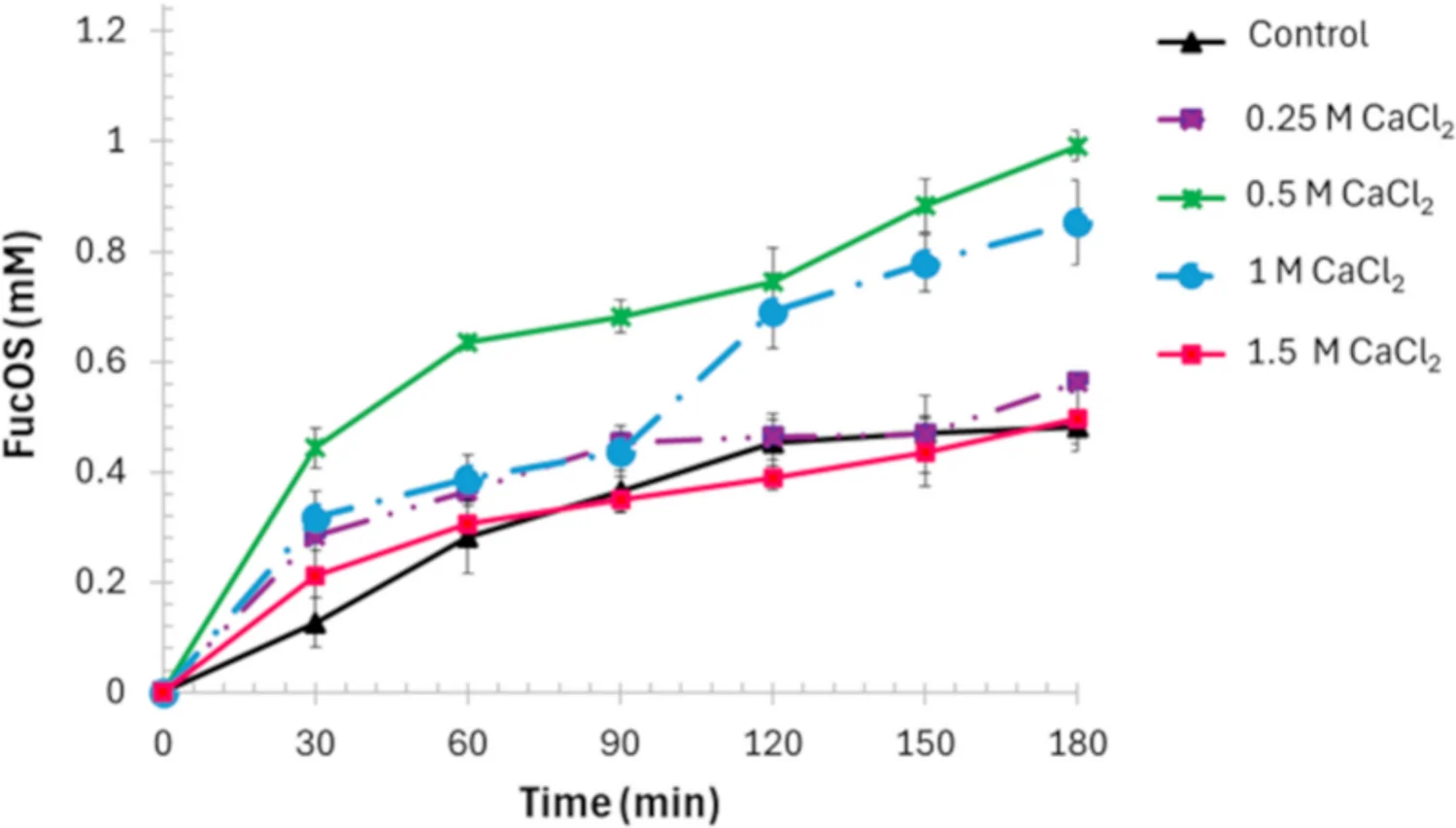
Transfucosylation kinetics catalyzed by FUC-*Tm* in the presence and absence of CaCl_2_. Reactions were conducted with 3.5 mM *p*NP-Fuc (donor) and 146 mM lactose (acceptor) in 1 mM acetate buffer (pH 5.0) at 60 °C for 3 h. Data are presented as mean ± standard deviation (*n* = 3)

As shown in Fig. 2, the presence of CaCl_2_ increased the transfucosylation activity of FUC-*Tm*, resulting in higher production of oligosaccharides. The largest increase in FucOS synthesis (106.13%) was observed at 0.5 M CaCl_2_ after 3 h of reaction, relative to the control.

To further evaluate the effect of Ca^2+^ on catalytic selectivity, the ratio between transfucosylation and hydrolysis rates (*V*_trans_/*V*_hydro_) was calculated by comparing the initial rates of both reactions. The transfucosylation rate (*V*_trans_) was determined from the initial rate of FucOS formation (mM/min), obtained from the linear region of the product concentration versus time curves. The hydrolysis rate (*V*_hydro_) was calculated from the initial rate of *p*NP release, determined spectrophotometrically at 410 nm using a standard calibration curve. The hydrolysis rate (*V*_hydro_) was determined under equivalent conditions in the absence of acceptor substrate. The ratio was calculated by dividing *V*_trans_ by *V*_hydro_. In the absence of Ca^2+^, the ratio was 0.223, whereas in the presence of 0.5 M CaCl_2_ it increased to 0.534, corresponding to a 2.39-fold increase. At CaCl_2_ concentrations above 0.5 M, the efficiency of FucOS synthesis decreased.

In addition, *a*_w_ decreased as the CaCl_2_ concentration increased (Table 1). The highest FucOS synthesis yield (55%) was observed at an *a*_w_ value of 0.976, corresponding to 0.5 M CaCl_2_. However, further decreases in *a*_w_ at higher CaCl_2_ concentrations did not result in additional increases in FucOS synthesis, indicating that the relationship between water activity and transfucosylation efficiency is not strictly monotonic under the conditions tested.

**Table 1:** Effect of water activity on FucOS synthesis at different CaCl_2_ concentrations

CaCl_2_ (M)	Activity water (*a*_w_)	Synthesis yield (%)^*^
0	0.995	21.64
0.25	0.987	34.21
0.5	0.976	55.24
1	0.952	44.67
1.5	0.920	38.40

^*^Yield calculated based on the hydrolyzed donor substrate

### Structural analysis of FUC-*Tm* in the presence of Ca^2+^ by molecular dynamics simulations

Based on the in vitro results, in which Ca^2+^ produced the greatest enhancement of FUC-*Tm* activity in both hydrolysis and transfucosylation reactions, its interaction with the enzyme was further investigated using MD simulations.

Two systems were modeled: the enzyme in the absence of Ca^2+^ (FUC-*Tm*) and the other in the presence of 0.5 M Ca^2+^ (FUC-*Tm-*Ca^2+^), corresponding to the concentration that yielded the highest FucOS synthesis in vitro. Three independent simulation replicas were performed to improve statistical robustness, and the trajectories were analyzed using multiple structural descriptors (see Supporting Information, Figs. S1–S6).

### Global structural stability

The root mean square deviation (RMSD) of Cα atoms was calculated to monitor the overall conformational stability of the enzyme during the simulations. RMSD values provide insight into large-scale structural deviations relative to the initial structure (Sargsyan et al. 2017).

The radius of gyration (*R*_g_) was also calculated to evaluate changes in the global compactness of the protein throughout the simulations (Gheibi et al. 2019). Previous studies have suggested that fluctuations exceeding ~ 2 Å in these parameters may indicate significant structural destabilization (Kato et al. 2017).

**Fig. 3:**
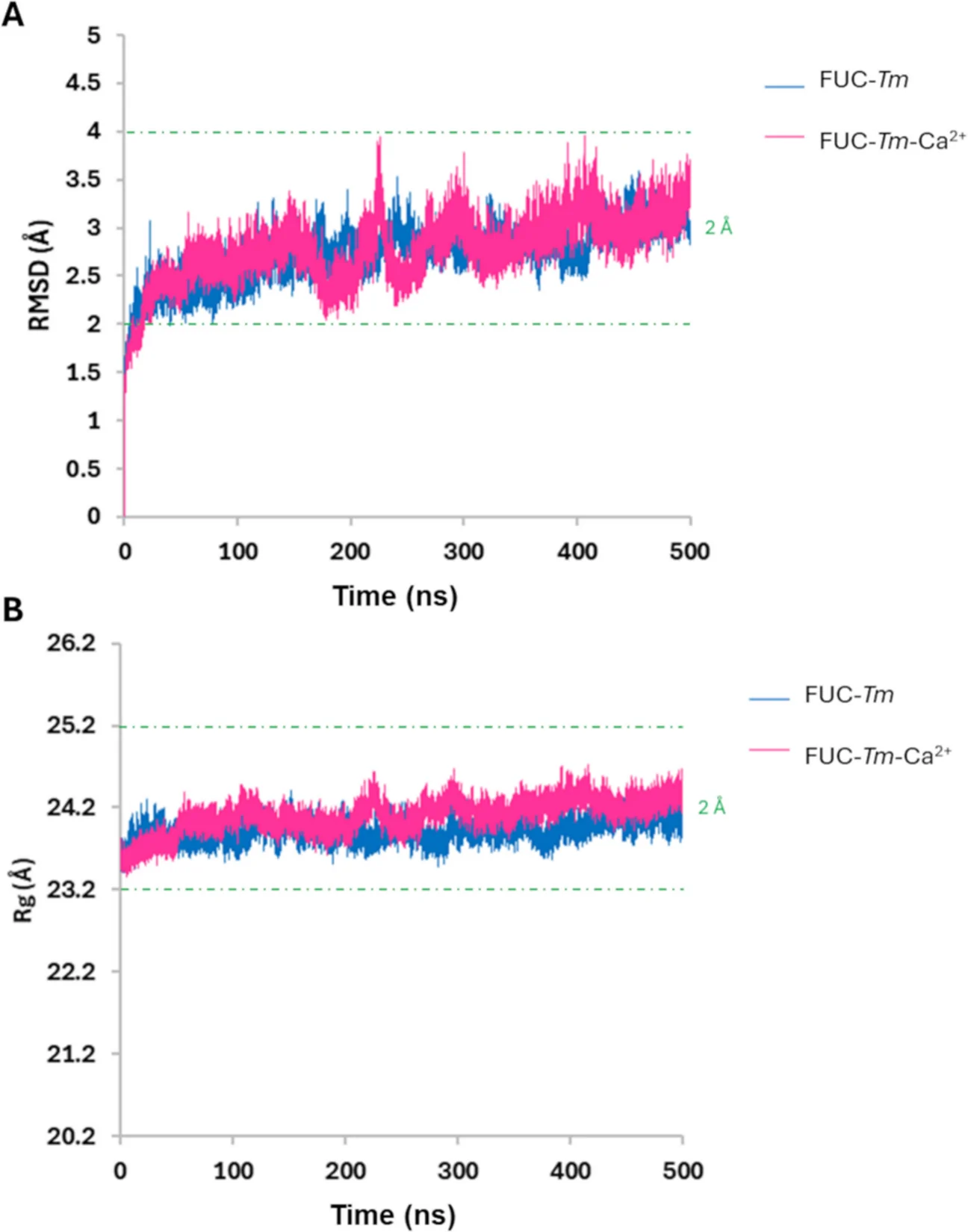
Global structural stability of FUC-*Tm* in the presence and absence of Ca^2+^ during molecular dynamics simulations. **A** Root-mean-square deviation (RMSD) of Cα atoms. **B** Radius of gyration (Rg) values for FUC-*Tm* (blue line) and FUC-*Tm*–Ca^2+^ systems (pink line)

As shown in Fig. 3A, RMSD values remained below 2 Å throughout the 500 ns simulations for both systems, indicating the absence of major conformational rearrangements. After approximately 300 ns, RMSD fluctuations decreased further and stabilized below 1 Å.

Similarly, the average *R*_g_ values were comparable for both systems, with mean values of 23.93 Å for FUC-*Tm* and 24.11 Å for FUC-*Tm*–Ca^2+^ (Fig. 3B), suggesting that the presence of Ca^2+^ does not significantly affect the global compactness of the enzyme.

### Local flexibility

Because no major global structural differences were detected, root mean square fluctuation (RMSF) analysis was performed to evaluate residue level flexibility. RMSF values describe the time-averaged positional fluctuations of amino acid residues relative to their Cα atoms and are widely used to identify flexible regions within proteins (Chen et al. 2016; Mahapatra et al. 2017; Liu et al. 2020).

**Fig. 4:**
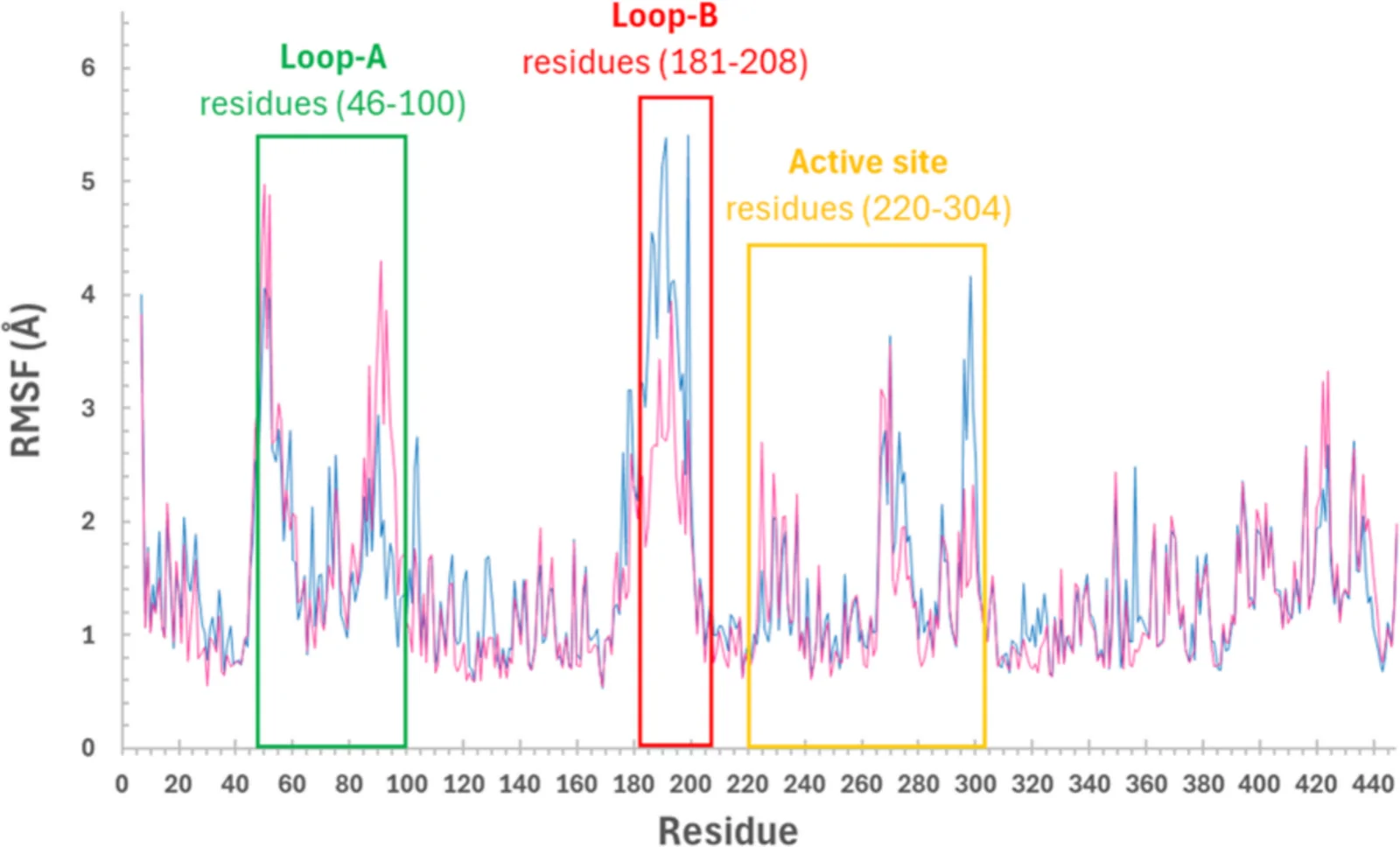
Residue-level flexibility of FUC-*Tm* in the absence and presence of Ca^2+^. RMSF profiles obtained from molecular dynamics simulations are shown for the FUC-*Tm* (blue line) and FUC-*Tm*–Ca^2+^ systems (pink line)

Figure 4 shows the averaged RMSF profiles obtained from simulations in the absence (blue line) and presence of Ca^2+^ (pink line). Three regions exhibited the highest flexibility across all replicas: Loop-A (residues 46–100), Loop-B (residues 181–208), and the active-site region (residues 220–304).

In the Ca^2+^-free system, Loop-A displayed moderate fluctuations exceeding 2 Å. In contrast, in the Ca^2+^-system, fluctuations in this region increased, reaching values of up to ~ 5 Å. However, trajectory inspection showed that this increase did not correspond to large-scale displacement of Loop-A away from the catalytic cleft.

Loop-B exhibited the opposite behavior. In the absence of Ca^2+^, RMSF values exceeded 5 Å, whereas in the presence of Ca^2+^, these fluctuations decreased to approximately 4 Å across all replicas.

Similarly, residues within the active-site region showed reduced RMSF values in the presence of Ca^2+^ (< 4 Å) compared with the Ca^2+^-free system (> 4 Å).

The spatial distribution of these fluctuations (Loop-A and Loop-B) can be visualized on the protein structure in the PDB representation shown in Figs. S7 and S8 (Supporting Information).

To further assess the dynamic behavior of the system, the kinetic energy was monitored throughout the simulations (Fig. S9). The Ca^2+^-system exhibited consistently lower kinetic energy values compared to the Ca^2+^-free system, suggesting reduced overall atomic mobility. This observation is consistent with the RMSF results, where decreased fluctuations were observed in Loop-B and the active-site region in the presence of Ca^2+^, supporting a more structurally stable catalytic environment.

### Active-site cavity analysis

**Fig. 5:**
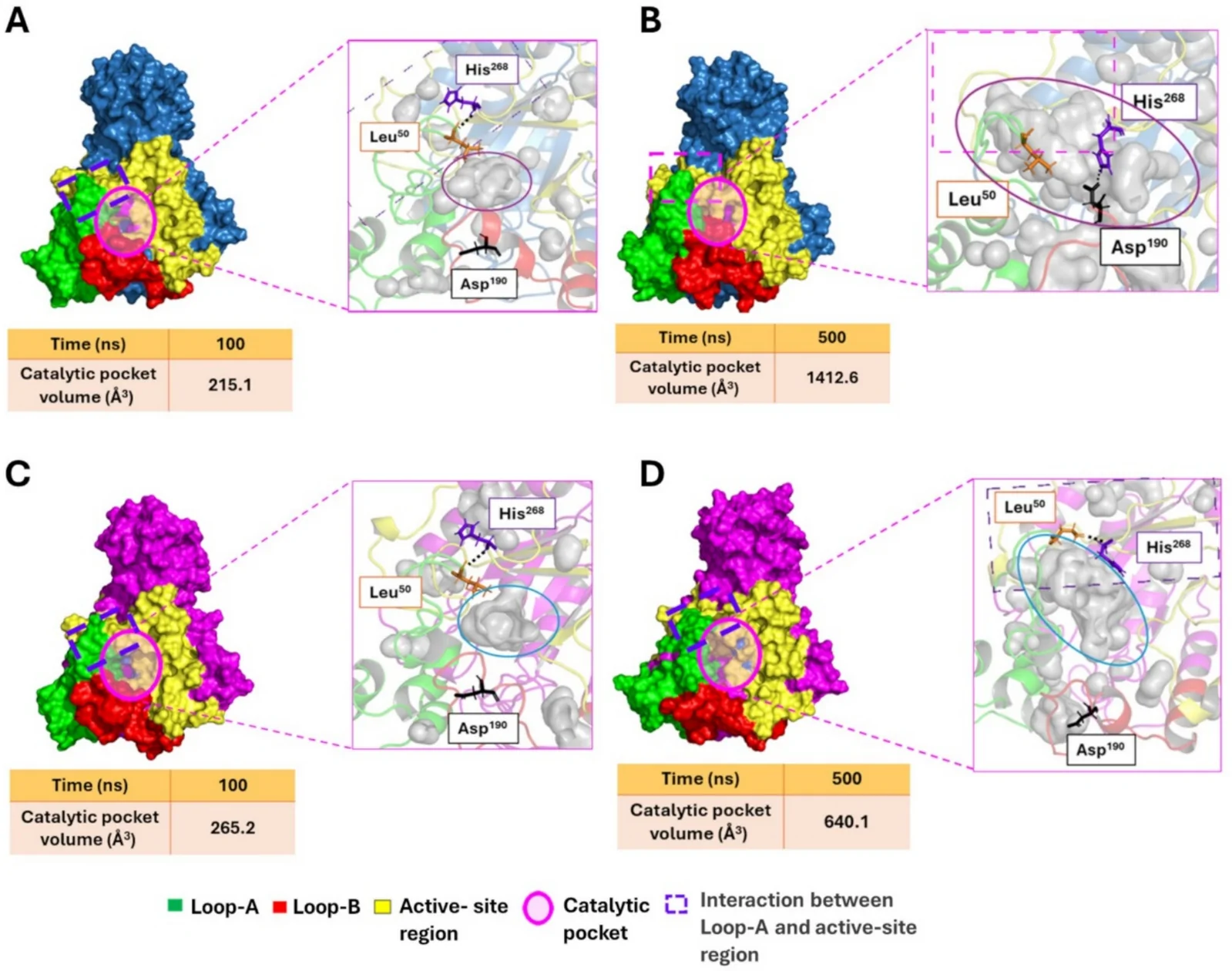
Structural changes in the active-site cavity of FUC-*Tm* during molecular dynamics simulations in the absence and presence of Ca^2+^. **A**, **B** Representative structures without Ca^2+^ at 100 ns and 500 ns, respectively. **C**, **D** Corresponding structures with Ca^2+^ at 100 ns and 500 ns, illustrating the spatial organization of catalytic residues and surrounding loop regions

To further evaluate the functional relevance of the fluctuations observed in Loops A and B and their impact on the architecture of the catalytic pocket, changes in the active-site cavity were analyzed along the MD simulations (Fig. 5).

As shown in Fig. 5A and B, in the absence of Ca^2+^ the volume of the catalytic cavity increased markedly during the simulation, expanding from 215.1 Å^3^ at 100 ns (Fig. 5A) to 1412.6 Å^3^ at 500 ns (Fig. 5B). An intermediate state at 303 ns showed a cavity volume of 636.5 Å^3^ (Fig. S10, Supporting Information). At the beginning of the trajectory, an interaction between Loop-A and the active-site region (His^268^–Leu^50^) contributed to the formation of a compact catalytic pocket. This interaction was progressively weakened over time, as observed in the intermediate structure (Fig. S10), and was completely lost at later stages of the simulation, resulting in a pronounced opening of the pocket and a substantial increase in cavity volume. This structural rearrangement led to the formation of a secondary adjacent cavity (highlighted by the pink dashed box). Concurrently, Loop-B approached the catalytic region (Fig. 5B). Additionally, Asp^190^ from Loop-B formed transient interactions with His^268^, displacing Leu^50^ and further contributing to the formation of the secondary cavity.

In contrast, in the presence of Ca^2+^ (Fig. 5C and D), the cavity volume increased more gradually during the simulation, from 265.2 Å^3^ at 100 ns (Fig. 5C) to 640.1 Å^3^ at 500 ns (Fig. 5D), with an intermediate value of 416.1 Å^3^ at 303 ns (Fig. S11, Supporting Information). Despite this increase, the cavity remained more compact compared to the Ca^2+^-free system and did not exhibit abrupt expansion. Notably, the interaction between Loop-A and the active-site region (His^268^–Leu^50^) was largely preserved throughout the simulation. Moreover, Loop-B displayed reduced conformational fluctuations, consistent with the RMSF analysis (Fig. 4), contributing to the maintenance of a more compact and structurally stable catalytic pocket.

### Molecular dynamics of the FUC-*Tm*–*p*NP-Fuc complex

MD simulations were performed for 100 ns to evaluate the stability and binding mode of the FUC-*Tm*–*p*NP-Fuc complex under Ca^2+^-free and Ca^2+^ presence. For each condition, three independent replicas were generated to improve statistical robustness.

Global structural stability and flexibility of the complex were first evaluated using RMSD, RMSF, and *R*_g_ analyses. Complete profiles are provided in the Supporting Information (Figs. S12–S17). The RMSD analysis shows that both systems reach a stable conformation after the equilibration phase. Although noticeable fluctuations were observed, particularly in the Ca^2+^-system, the RMSD values remained within a stable range without evidence of continuous drift, indicating overall structural stability of the system.

RMSF analysis revealed greater residue-level mobility in several regions of the Ca^2+^-free system, particularly in solvent-exposed loops. In contrast, the presence of Ca^2+^ was associated with reduced residue flexibility. Consistently, the *R*_g_ remained relatively constant in both systems, although the Ca^2+^-system simulations showed lower dispersion among replicate trajectories, suggesting a more stable conformational ensemble.

Δ*G*ᵤ were estimated using the MM/PBSA method. The average Δ*G*ᵤ for the complex in the absence of Ca^2+^ was −16.5 kJ/mol, whereas in the presence of Ca^2+^ it decreased to −19.56 kJ/mol, indicating stronger enzyme–substrate interactions in the Ca^2+^-system.

To further evaluate the catalytic relevance of the binding mode, the docking-derived conformation of the FUC-*Tm*–*p*NP-Fuc complex was compared with the crystal structure of FUC-*Tm* bound to fucose (PDB ID: 1ODU) (Sulzenbacher et al. 2004).

**Fig. 6:**
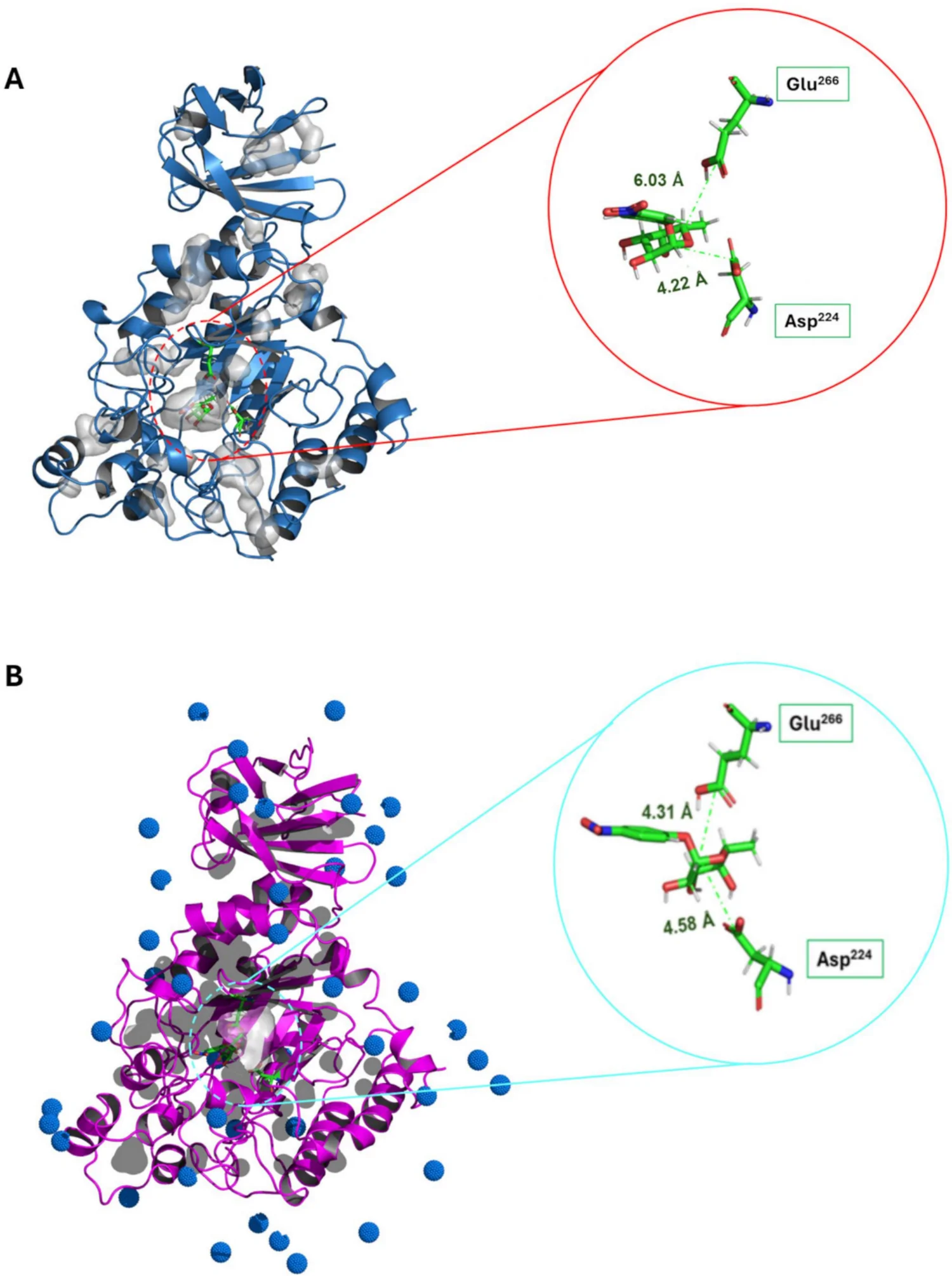
Structural representation of the FUC-*Tm*–*p*NP-Fuc complex. **A** System without Ca^2+^. **B** System with Ca^2+^ (blue dots). Circles highlight the distances between catalytic residues (Asp^224^, Glu^266^) and the C1 carbon of fucose within the *p*NP-Fuc substrate

In the Ca^2+^-free simulation (Fig. 6A), the catalytic residue Asp^224^ was located 4.2 Å from the anomeric carbon (C1) of the fucose moiety in *p*NP-Fuc, whereas Glu^266^ was positioned at a distance of 6.03 Å.

In contrast, in the Ca^2+^-system simulation (Fig. 6B), both catalytic residues remained within approximately 4 Å of the fucose C1 carbon, a distance consistent with catalytically competent configurations reported for α-L-fucosidases.

## Discussion

### Effect of divalent ions on hydrolytic activity

The results demonstrate that the activating effect of divalent ions on FUC-*Tm* depends strongly on the specific physicochemical properties of each ion. Differences in ionic radius, charge density, hydration shell size, and coordination geometry are known to influence the interaction between metal ions and protein surfaces, thereby affecting enzyme conformation and stability (Jahnen-Dechent and Ketteler 2012; Dudev and Lim 2013). These parameters likely determine the extent to which each ion modulates the catalytic behavior of FUC-*Tm*.

The decrease in enzymatic activity observed at higher salt concentrations may be associated with increased ionic strength in the reaction medium. Elevated ionic strength can alter electrostatic interactions within the protein and between the enzyme and its substrate, potentially leading to subtle conformational perturbations or reduced catalytic efficiency (Arroyo 1998; Fried et al. 2013). Such effects may explain the decline in hydrolytic activity observed at the highest ion concentrations tested.

Similar ion-dependent activation phenomena have been reported for other α-L-fucosidases. For example, Liu et al. (2016) reported activity increases of 50% and 56% for the α-L-fucosidases Eo0918 and Eo3812 from *Emticicia oligotrophica* in the presence of Ca^2+^. Likewise, Bishnoi et al. (2018) observed that the α-L-fucosidase from *Streptosporangium roseum* exhibited activity increases of 93% and 7% in the presence of Ca^2+^ and Mn^2+^, respectively. More recently, Liu et al. (2022) reported moderate activity enhancements in the α-L-fucosidase from *Pseudoalteromonas* sp. OUO3 in the presence of Mg^2+^ and Mn^2+^. Collectively, these studies indicate that divalent cations can significantly influence catalytic performance in α-L-fucosidases, although the underlying molecular mechanisms remain incompletely understood.

### Effect of calcium on transfucosylation activity

In addition to enhancing hydrolytic activity, Ca^2+^ also increased the relative contribution of transfucosylation, as reflected by the higher *V*_trans_/*V*_hydro_ ratio. This observation suggests that Ca^2+^ partially shifts the catalytic balance toward oligosaccharide synthesis under appropriate reaction conditions.

A comparable effect was previously reported by Guzmán-Rodríguez et al. (2018), who observed an approximately 120% increase in FucOS production by FUC-*Tm* in the presence of 1.1 M CaCl_2_. Together, these findings support the notion that Ca^2+^ positively modulates the catalytic behavior of FUC-*Tm* and promotes transfucosylation under specific physicochemical conditions.

However, the effect of metal ions on glycosidase-catalyzed transglycosylation is complex and often concentration-dependent. For example, Arsov et al. (2022) reported a modest increase in galactooligosaccharide production when Mg^2+^ was added to a β-galactosidase system, whereas higher concentrations resulted in inhibitory effects attributed to increased ionic strength. A similar trend was observed in the present study, where CaCl_2_ concentrations above 0.5 M reduced transfucosylation efficiency.

Changes in *a*_w_ may also contribute to these observations. Transglycosylation reactions catalyzed by glycosidases are generally favored at reduced water availability, whereas hydrolysis becomes dominant when water is abundant. This interpretation is consistent with previous reports showing that reduced water activity promotes transfucosylation in FUC-*Tm* (Robles-Arias et al. 2021). However, the results obtained in this study indicate that this relationship is not strictly monotonic under the conditions evaluated. While a decrease in *a*_w_ from 0.995 to 0.976 (0.5 M CaCl_2_) correlates with increased FucOS synthesis, further reduction in *a*_w_ at higher CaCl_2_ concentrations (e.g., 0.920 at 1.5 M) does not lead to additional improvement and instead coincides with increased hydrolytic activity. Further simulations at higher ionic strengths would be required to establish a direct structural basis for this inhibitory effect.

This behavior suggests that the effect of CaCl_2_ is governed by a balance between reduced water availability and increased ionic strength. At moderate concentrations, the decrease in aw may favor transglycosylation, whereas at higher concentrations, elevated ionic strength may alter enzyme–substrate interactions and catalytic efficiency, shifting the balance toward hydrolysis. Therefore, the observed catalytic behavior reflects the combined influence of physicochemical and structural factors rather than water activity alone.

Previous computational studies have shown that metal ions can influence the availability and orientation of catalytic water molecules within the active site, thereby affecting the balance between hydrolysis and glycosyl transfer reactions (Brás et al. 2010). These findings may explain why maximal hydrolytic activity was observed at higher CaCl_2_ concentrations, whereas optimal FucOS synthesis occurred at moderate Ca^2+^ levels. Under these conditions, Ca^2+^ may promote stabilization of catalytic intermediates and facilitate productive substrate binding without excessively favoring hydrolysis. Together, these effects likely contribute to the observed shift toward transfucosylation.

Importantly, the increase in the *V*_trans_/*V*_hydro_ ratio cannot be attributed solely to structural effects observed in MD simulations. The observed shift is more likely influenced by physicochemical factors, particularly the decrease in *a*_w_, which reduces the availability of water as a competing nucleophile and favors transglycosylation. Therefore, the enhanced transfucosylation efficiency is best explained by the combined contribution of structural stabilization and physicochemical effects associated with reduced *a*_w_.

It should be noted that the experimental design does not allow for a clear separation between the effects of reduced water activity and those associated with the presence of Ca^2+^. Therefore, the contribution of each factor to the observed increase in the *V*_trans_/*V*_hydro_ ratio cannot be independently quantified in this study.

### Structural effects of Ca^2+^ on FUC-*Tm* revealed by molecular dynamics simulations

MD simulations indicate that Ca^2+^ does not induce large-scale conformational changes in FUC-*Tm*. Instead, its effect appears to involve localized modulation of structural dynamics in regions that are functionally relevant for catalysis. The RMSD profiles obtained from independent trajectories showed comparable global structural stability in the presence and absence of Ca^2+^, suggesting that the ion does not significantly alter the overall fold of the enzyme. Similar behavior has been reported by Gheibi et al. (2019), who observed that Ca^2+^ and Na^+^ ions did not induce significant global structural changes in the A8 and A9 subunits of calprotectin, as evidenced by RMSD values below 2 Å.

In contrast, residue-level analyses revealed pronounced differences in local flexibility. RMSF analysis showed that the presence of Ca^2+^ reduces fluctuations in Loop-B and in residues surrounding the catalytic pocket. Regions displaying RMSF values between 4 and 6 Å are typically associated with substantial conformational mobility (Liu et al. 2020), and the reduction in flexibility observed here suggests that Ca^2+^ stabilizes structural elements adjacent to the catalytic site. The reduction in Loop-B flexibility is associated with a more defined catalytic geometry, which may enhance overall catalytic efficiency. However, this structural effect alone does not explain the preferential increase in transfucosylation over hydrolysis.

At first glance, the increased flexibility of Loop-A appears inconsistent with its proposed role in maintaining the His^268^–Leu^50^ interaction required for active-site formation. However, RMSF reflects the amplitude of local motions and does not distinguish between productive fluctuations near the catalytic cleft and large-scale displacement away from it. Trajectory inspection showed that, in the absence of Ca^2+^, Loop-A displayed lower average RMSF values but occasionally sampled conformations in which the His^268^–Leu^50^ interaction was disrupted. Although this interaction has been suggested to contribute to active-site organization, its functional role may not require rigid stabilization under all conditions. Instead, transient fluctuations in Loop-A may enable dynamic rearrangements compatible with catalytic activity. These events coincided with pronounced expansion of the active-site pocket and the formation of a secondary cavity. By contrast, in the presence of Ca^2+^, Loop-A exhibited higher RMSF values, reaching up to ~ 5 Å, but remained spatially confined near the catalytic cleft. Interestingly, rather than representing a destabilizing effect, this increased flexibility may reflect a functional differentiation in loop dynamics, where reduced flexibility in Loop-B contributes to maintaining catalytic geometry, while increased mobility in Loop-A may facilitate conformational adaptability required for substrate access or product release. Simultaneously, Loop-B became less mobile, and the active-site region appeared more stable.

Thus, Ca^2+^ appears to redistribute flexibility within the catalytic pocket: Loop-B and the surrounding residues become more rigid, whereas Loop-A retains greater local mobility within a spatially confined conformation. In this context, Loop-B may act as a sensitive indicator of the local structural changes induced by Ca^2+^, rather than as an isolated determinant of catalytic behavior.

Consistent with these observations, analysis of the active-site cavity revealed that, in the absence of Ca^2+^, the catalytic pocket undergoes substantial expansion during the simulation, associated with disruption of the His^268^–Leu^50^ interaction and the formation of a secondary cavity. In contrast, in the presence of Ca^2+^, the cavity expansion is more gradual and remains comparatively limited, while the His^268^–Leu^50^ interaction is largely preserved.

In addition, the analysis of kinetic energy throughout the simulations revealed consistently lower values in the Ca^2+^-system, indicating reduced overall atomic mobility. This observation is consistent with the RMSF results and supports the notion of a more globally stabilized dynamic state in the presence of Ca^2+^. Together with the reduced cavity expansion, these findings provide converging evidence that Ca^2+^ promotes a more controlled and structurally stable catalytic environment.

This stabilization may help maintain an optimal geometry of the catalytic cavity by preventing excessive expansion of the active-site pocket. Rather than reflecting a specific interaction with individual residues, this effect is more consistent with a globally stabilized catalytic environment. Such stabilization could facilitate substrate positioning and preserve a catalytically competent geometry, consistent with the experimentally observed increase in both hydrolytic and transfucosylation activities. However, the present simulations do not directly demonstrate that the reduced cavity size selectively favors transfucosylation over hydrolysis.

Although reduced fluctuations were observed in Loop-B and surrounding residues in the presence of Ca^2+^, the present simulations do not provide direct evidence of a specific Ca^2+^-binding site. Therefore, the observed stabilization is more consistent with a global electrostatic effect and ionic strength–mediated modulation of protein dynamics rather than residue-specific coordination. The high concentration of CaCl_2_ used in the simulations likely contributes to this effect by altering the electrostatic environment of the protein.

Recent computational studies have suggested that catalytic efficiency in glycosidases is closely linked to the structural stability and geometry of the catalytic pocket and that these factors may also influence transglycosylation performance. Excessive expansion or destabilization of this region can impair productive substrate binding and promote nonproductive interactions (Jitonnom et al. 2025).

In agreement with this concept, the present results suggest that Ca^2+^ contributes to the stabilization of flexible regions adjacent to the active site, thereby maintaining a controlled cavity expansion compatible with efficient catalysis. However, the available data do not allow us to conclude that this effect selectively favors transfucosylation through a direct effect on Loop-B. Instead, the higher transfucosylation/hydrolysis ratio is more likely explained by the combined contribution of global active-site stabilization and reduced *a*_w_, which decreases the availability of water as a competing nucleophile.

Comparable effects have been reported in other glycosidases. For instance, Hou et al. (2023) demonstrated that Mn^2+^ reduces fluctuations in flexible loop regions of a xylanase, leading to improved catalytic pocket geometry and enhanced substrate accessibility. Together, these findings support the hypothesis that metal-induced stabilization of loop regions may represent a general mechanism by which divalent ions modulate glycosidase activity.

### Molecular dynamics of the FUC-*Tm*–substrate complex

The simulations of the FUC-*Tm*–*p*NP-Fuc complex further support the stabilizing effect of Ca^2+^ on the enzyme–substrate interactions. Lower RMSD values and reduced residue-level fluctuations in the Ca^2+^-system suggest the formation of a more stable enzyme–substrate complex. Likewise, the reduced dispersion in *R*_g_ values indicates the presence of a more conformationally consistent structural ensemble in the Ca^2+^-system trajectories.

Energetic analysis using MM/PBSA calculations provided additional support for this interpretation. The more negative binding free energy (Δ*G*_u_) obtained in the presence of Ca^2+^ suggests stronger enzyme–substrate interactions relative to the Ca^2+^-free system. This energetic trend is consistent with the reduced flexibility of Loop-B and the controlled expansion of the catalytic pocket observed in the structural analyses.

In the absence of Ca^2+^, the larger catalytic cavity may permit non-productive substrate orientations that weaken binding interactions and reduce catalytic efficiency. In contrast, Ca^2+^ appears to promote a more favorable catalytic geometry. This interpretation is further supported by the analysis of catalytic residue distances. In the Ca^2+^-free simulations, the distance between Glu^266^ and the anomeric carbon frequently exceeded the optimal catalytic distance (~ 4.1 Å) reported for α-L-fucosidases (Sulzenbacher et al. 2004; Pavón-Chimal et al. 2022). In contrast, the Ca^2+^-system maintained both catalytic residues within catalytically relevant distances, consistent with a configuration favorable for productive catalysis.

Taken together, these results indicate that Ca^2+^ is associated with the stabilization of a catalytically competent environment through modulation of protein dynamics and electrostatic conditions. However, the present data do not provide evidence of a specific Ca^2+^-binding interaction or direct residue coordination. Instead, the enhanced transfucosylation/hydrolysis ratio is more likely explained by the combined contribution of global active-site stabilization, reduced water activity (*a*_w_), and a lower overall dynamic (energetic) state of the system.

Overall, the results of this study indicate that the effect of Ca^2+^ on the catalytic behavior of FUC-*Tm* cannot be attributed to a single dominant mechanism. Instead, the observed changes arise from the combined influence of structural and physicochemical factors. Molecular dynamics simulations suggest that the presence of Ca^2+^ is associated with localized modulation of protein dynamics, particularly in loop regions surrounding the catalytic pocket, which may contribute to maintaining a catalytically competent geometry. At the same time, experimental data indicate that variations in water activity and ionic strength play a significant role in modulating reaction selectivity, particularly by influencing the balance between hydrolysis and transfucosylation. Therefore, the catalytic response of FUC-*Tm* to Ca^2+^ should be understood as a multifactorial process in which structural dynamics and environmental conditions act together to determine enzyme performance.

## Conclusions

In this study, we investigated the influence of divalent cations on the catalytic behavior of the α-L-fucosidase from *Thermotoga maritima* (FUC-*Tm*) through a combined experimental and computational approach. The results demonstrate that Ca^2+^ enhances both hydrolytic and transfucosylation activities under specific conditions, with the highest transfucosylation efficiency observed at intermediate CaCl_2_ concentrations. Molecular dynamics simulations suggest that the presence of Ca^2+^ is associated with reduced flexibility in loop regions surrounding the catalytic pocket, which may contribute to maintaining a geometry favorable for enzyme–substrate interactions. However, no specific Ca^2+^ binding site or defined coordination pattern was identified, and the observed structural effects are interpreted as arising from a combination of nonspecific electrostatic interactions and transient ion–protein contacts. Importantly, the enhancement in catalytic selectivity cannot be attributed solely to the presence of Ca^2+^, as changes in water activity and ionic strength also play a significant role. Therefore, the observed behavior reflects a balance between structural and physicochemical factors. Overall, this work provides a more comprehensive framework for understanding ion-mediated modulation of glycoside hydrolases and highlights the importance of considering both molecular dynamics and environmental conditions in the design of improved biocatalytic systems.

## Supplementary Information

Below is the link to the electronic supplementary material.

ESM 1 Supplementary Material 1 (PDF 1.11 MB)

## Data Availability

All data supporting the findings of this study are available within the article and its Supplementary Information.

## References

[CR1] Abraham MJ, Murtola T, Schulz R, Páll S, Smith JC, Hess B, Lindahl E (2015) GROMACS: high performance molecular simulations through multi-level parallelism from laptops to supercomputers. SoftwareX 1–2:19–25. 10.1016/j.softx.2015.06.001

[CR2] Arroyo M (1998) Inmobilized enzymes: theory, methods of study and applications. Ars Pharm 39:23–29. 10.30827/ars.v39i2.6008

[CR3] Arsov A, Ivanov I, Tsigoriyna L, Petrov K, Petrova P (2022) In vitro production of galactooligosaccharides by a novel β-galactosidase of *Lactobacillus bulgaricus*. Int J Mol Sci 23:14308. 10.3390/ijms232214308 36430784 10.3390/ijms232214308 PMC9697242

[CR4] Berman HM, Westbrook J, Feng Z, Gilliland G, Bhat TN, Weissig H, Shindyalov IN, Bourne PE (2000) The Protein Data Bank. Nucleic Acids Res 28:235–242. 10.1093/nar/28.1.235 10592235 10.1093/nar/28.1.235 PMC102472

[CR5] BIOVIA, Dassault Systèmes (2021) Discovery studio visualizer, version 20.1.0.19295. Dassault Systèmes

[CR6] Bishnoi R, Mahajan S, Ramya TNC (2018) An F-type lectin domain directs the activity of *Streptosporangium roseum* alpha-L-fucosidase. Glycobiology 28:860–875. 10.1093/glycob/cwy079 30169639 10.1093/glycob/cwy079 PMC6455950

[CR7] Brás NF, Fernandes PA, Ramos MJ (2010) QM/MM studies on the β-galactosidase catalytic mechanism: hydrolysis and transglycosylation reactions. J Chem Theory Comput 6:421–433. 10.1021/ct900530f 26617299 10.1021/ct900530f

[CR8] Bridiau N, Issaoui N, Maugard T (2010) The effects of organic solvents on the efficiency and regioselectivity of N‐acetyl‐lactosamine synthesis, using the β‐galactosidase from *Bacillus circulans* in hydro‐organic media. Biotechnol Prog 26:1278–1289. 10.1002/btpr.445 20568279 10.1002/btpr.445

[CR9] Chen J, Wang J, Zhu W (2016) Molecular mechanism and energy basis of conformational diversity of antibody SPE7 revealed by molecular dynamics simulation and principal component analysis. Sci Rep 6:1–10. 10.1038/srep36900 27830740 10.1038/srep36900 PMC5103278

[CR10] Coines J, Cuxart I, Teze D, Rovira C (2022) Computer simulation to rationalize “rational” engineering of glycoside hydrolases and glycosyltransferases. J Phys Chem B 126:802–812. 10.1021/acs.jpcb.1c09536 35073079 10.1021/acs.jpcb.1c09536 PMC8819650

[CR11] de Sanctis D, Inácio JM, Lindley PF, de Sá-Nogueira I, Bento I (2010) New evidence for the role of calcium in the glycosidase reaction of GH43 arabinanases. FEBS J 277:4562–4574. 10.1111/j.1742-4658.2010.07870.x 20883454 10.1111/j.1742-4658.2010.07870.x

[CR12] Dudev T, Lim C (2013) Competition among metal ions for protein binding sites: determinants of metal ion selectivity in proteins. Chem Rev 114:538–556. 10.1021/cr4004665 24040963 10.1021/cr4004665

[CR13] Fried SD, Wang L, Boxer SG, Ren P, Pande VS (2013) Calculations of the electric fields in liquid solutions. J Phys Chem B 117:16236–16248. 10.1021/jp410720y 24304155 10.1021/jp410720y PMC4211882

[CR14] Frisch M, Trucks G, Schlegel H, Scuseria G, Robb M, Cheeseman J, Scalmani G, Barone V, Petersson G, Nakatsuji H, Caricato M, Li X, Hratchian H, Izmaylov A, Bloino J, Zheng G, Sonnenberg J, Hada M, Ehara M, Fox D (2016) Gaussian 09, Revision A.02. Gaussian inc., Wallingford CT

[CR15] Gheibi N, Ghorbani M, Shariatifar H, Farasat A (2019) In silico assessment of human calprotectin subunits (S100A8/A9) in presence of sodium and calcium ions using molecular dynamics simulation approach. PLoS One 14:e0224095. 10.1371/journal.pone.0224095 31622441 10.1371/journal.pone.0224095 PMC6797115

[CR16] Grace Development Team (n.d.) Grace – WYSIWYG 2D plotting tool. Available at: http://plasma-gate.weizmann.ac.il/Grace/. Accessed 5 Mar 2024

[CR17] Guzmán-Rodríguez F, Alatorre-Santamaría S, Gómez-Ruiz L, Rodríguez-Serrano G, García-Garibay M, Cruz-Guerrero AE (2018) Improvement of the transfucosylation activity of α-L-fucosidase from *Thermotoga maritima* for the synthesis of fucosylated oligosaccharides in the presence of calcium and sodium. Extremophiles 22:889–894. 10.1007/s00792-018-1045-4 30088105 10.1007/s00792-018-1045-4

[CR18] Hou M, Liang C, Fei Y, Yang D, Zhang N, Lu Y, Wang L, Xing Z, Zhao Z (2023) Analysis of the effect of metal ions on the ability of xylanase to hydrolyze wheat bran by molecular dynamics simulations. Front Bioeng Biotechnol 11:1–12. 10.3389/fbioe.2023.1142873 10.3389/fbioe.2023.1142873 PMC9978823 36873368

[CR19] Humphrey W, Dalke A, Schulten K (1996) VMD: visual molecular dynamics. J Mol Graph 14:33–38. 10.1016/0263-7855(96)00018-5 8744570 10.1016/0263-7855(96)00018-5

[CR20] Jahnen-Dechent W, Ketteler M (2012) Magnesium basics. Clin Kidney J 5:i3–i14. 10.1093/ndtplus/sfr163 26069819 10.1093/ndtplus/sfr163 PMC4455825

[CR21] Jitonnom W, Wanjai T, Friedman R, Jitonnom J (2025) Mechanistic insights and computer‐informed design of α‐galactosidase for galactooligosaccharide synthesis. ChemCatChem 17:22. 10.1002/cctc.202501207

[CR22] Kato K, Nakayoshi T, Fukuyoshi S, Kurimoto E, Oda A (2017) Validation of molecular dynamics simulations for prediction of three-dimensional structures of small proteins. Molecules 22:1716. 10.3390/molecules22101716 29023395 10.3390/molecules22101716 PMC6151455

[CR23] Kim S, Chen J, Cheng T, Gindulyte A, He J, He S, Li Q, Shoemaker BA, Thiessen PA, Yu B, Zaslavsky L, Zhang J, Bolton EE (2023) PubChem 2023 update. Nucleic Acids Res 51:D1373–D1380. 10.1093/nar/gkac956 36305812 10.1093/nar/gkac956 PMC9825602

[CR24] Klontz EH, Li C, Kihn K et al (2020) Structure and dynamics of an α-fucosidase reveal a mechanism for highly efficient IgG transfucosylation. Nat Commun 11:6204. 10.1038/s41467-020-20044-z 33277506 10.1038/s41467-020-20044-z PMC7718225

[CR25] Kobayashi M, Hondoh H, Suzuki M, Saburi W, Mori H, Okuyama M, Kimura A (2011) Calcium ion-dependent increase in thermostability of dextran glucosidase from *Streptococcus mutans*. Biosci Biotechnol Biochem 75:1557–1563. 10.1271/bbb.110256 21821929 10.1271/bbb.110256

[CR26] Krupinskaitė A, Stanislauskienė R, Serapinas P, Rutkienė R, Gasparavičiūtė R, Meškys R, Stankevičiūtė J (2024) α-L-fucosidases from an alpaca faeces metagenome: characterisation of hydrolytic and transfucosylation potential. Int J Mol Sci 25:809. 10.3390/ijms25020809 38255883 10.3390/ijms25020809 PMC10815079

[CR27] Lee J, Cheng X, Swails JM, Yeom MS, Eastman PK, Lemkul JA, Wei S, Buckner J, Jeong JC, Qi Y, Jo S, Pande VS, Case DA, Brooks CL III, MacKerell AD Jr, Klauda JB, Im W (2016) CHARMM-GUI input generator for NAMD, GROMACS, AMBER, OpenMM, and CHARMM/OpenMM simulations using the CHARMM36 additive force field. J Chem Theory Comput 12:405–413 26631602 10.1021/acs.jctc.5b00935 PMC4712441

[CR28] Liu S, Kulinich A, Cai ZP, Hong YM, Du YM, Yong ML, Liu L, Voglmeir J (2016) The fucosidase-pool of *Emticicia oligotrophica*: biochemical characterization and transfucosylation potential. Glycobiology 26:871–879. 10.1093/glycob/cww030 26941394 10.1093/glycob/cww030

[CR29] Liu P, Zhang H, Wang Y, Chen X, Jin L, Xu L, Xiao M (2020) Screening and characterization of an α-L-fucosidase from *Bacteroides fragilis* NCTC9343 for synthesis of fucosyl-N-acetylglucosamine disaccharides. Appl Microbiol Biotechnol 104:7827–7840. 10.1007/s00253-020-10759-w 32715363 10.1007/s00253-020-10759-w

[CR30] Liu X, Geng X, Liu W, Lyu Q (2022) Biochemical characterization of an α-fucosidase *Psa*Fuc from the GH29 family. Process Biochem 122:258–266. 10.1016/j.procbio.2022.09.004

[CR31] Mahapatra MK, Bera K, Singh DV, Kumar R, Kumar M (2017) *In silico* modelling and molecular dynamics simulation studies of thiazolidine based PTP1B inhibitors. J Biomol Struct Dyn 36:1195–1211. 10.1080/07391102.2017.1317026 28393626 10.1080/07391102.2017.1317026

[CR32] Meng EC, Pettersen EF, Couch GS, Huang CC, Ferrin TE (2006) Tools for integrated sequence-structure analysis with UCSF Chimera. BMC Bioinformatics 7:339. 10.1186/1471-2105-7-339 16836757 10.1186/1471-2105-7-339 PMC1570152

[CR33] Morris G, Huey R, Lindstrom W, Sanner M, Belew R, Goodsell D, Oson A (2009) AutoDock4 and AutoDockTools4: automated docking with selective receptor flexibility. J Comput Chem 30:2785–2791. 10.1002/jcc.21256.AutoDock4 19399780 10.1002/jcc.21256 PMC2760638

[CR34] Moya-Gonzálvez EM, Zeuner B, Thorhallsson AT, Holck J, Palomino-Schätzlein M, Rodríguez-Díaz J, Meyer AS, Yebra MJ (2024) Synthesis of fucosyllactose using α-L-fucosidases GH29 from infant gut microbial metagenome. Appl Microbiol Biotechnol 108:338. 10.1007/s00253-024-13178-3 38771321 10.1007/s00253-024-13178-3 PMC11108932

[CR35] Pavón-Chimal ME, Jiménez-Pérez C, Guzmán-Rodriguez F, Alatorre-Santamaría S, González-Olivares LG, García-Garibay M, Gómez-Ruiz L, Rodríguez-Serrano G, Cruz-Guerrero AE (2022) Mechanistic insight into the synthesis of fucooligosaccharides by α-L-fucosidase from *Thermotoga maritima* belonging to the GH29 family: *in silico* study. Biologia Bratisl 78:1825–1832. 10.1007/s11756-022-01296-0

[CR36] Perdew JP, Burke K, Ernzerhof M (1996) Generalized gradient approximation made simple. Phys Rev Lett 77:3865–3868. 10.1103/physrevlett.77.3865 10062328 10.1103/PhysRevLett.77.3865

[CR37] Pérez-Escalante E, González-Olivares LG, Castañeda-Ovando A, Cruz-Guerrero AE, Trant JF, López-Orozco W, Mendoza-Huizar LH, Alatorre-Santamaría S (2021) An in silico approach to enzymatic synthesis of fucooligosaccharides using α-L-fucosidase from *Thermotoga maritima*. Chem Proc 3:10. 10.3390/ecsoc-24-08303

[CR38] Robles-Arias MA, García-Garibay M, Alatorre-Santamaría S, Tello-Solís SR, Guzmán-Rodriguez F, Gómez-Ruiz L, Rodríguez-Serrano G, Cruz-Guerrero AE (2021) Improvement of fucosylated oligosaccharides synthesis by α-L-fucosidase from *Thermotoga maritima* in water-organic cosolvent reaction system. Appl Biochem Biotechnol 193:3553–3569. 10.1007/s12010-021-03628-3 34312785 10.1007/s12010-021-03628-3

[CR39] Robles-Arias MA, Jiménez-Pérez C, Gómez-Castro CZ, Alatorre-Santamaría S, Guzmán-Rodríguez F, García-Garibay M, Cruz-Guerrero AE (2025) Effect of temperature on the structure of α-L-fucosidase from *Thermotoga maritima*: implications from molecular dynamics simulation. J Biomol Struct Dyn 1–15. 10.1080/07391102.2025.2543365 10.1080/07391102.2025.2543365 40794893

[CR40] Rudloff S, Kunz C (2012) Milk oligosaccharides and metabolism in infants. Adv Nutr 3(3):398S-405S. 10.3945/an.111.001594 22585918 10.3945/an.111.001594 PMC3649476

[CR41] Sargsyan K, Grauffel C, Lim C (2017) How molecular size impacts RMSD applications in molecular dynamics simulations. J Chem Theory Comput 13:1518–1524. 10.1021/acs.jctc.7b00028 28267328 10.1021/acs.jctc.7b00028

[CR42] Schrödinger LLC (2015) The PyMOL molecular graphics system, version 2.4.0. Schrödinger LLC

[CR43] Sulzenbacher G, Bignon C, Nishimura T, Tarling CA, Withers SG, Henrissat B, Bourne Y (2004) Crystal structure of *Thermotoga maritima* α-l-Fucosidase. J Biol Chem 279:13119–13128. 10.1074/jbc.m313783200 14715651 10.1074/jbc.M313783200

[CR44] Valdés-Tresanco MS, Valdés-Tresanco ME, Valiente PA, Moreno E (2021) gmx_MMPBSA: a new tool to perform end-state free energy calculations with GROMACS. J Chem Theory Comput 17:6281–6291. 10.1021/acs.jctc.1c00645 34586825 10.1021/acs.jctc.1c00645

[CR45] Vera C, Guerrero C, Wilson L, Illanes A (2017) Optimization of reaction conditions and the donor substrate in the synthesis of hexyl-β-d-galactoside. Process Biochem 58:128–136. 10.1016/j.procbio.2017.05.005

[CR46] Wilkens C, Vuillemin M, Pilgaard B, Polikarpovb I, Mortha JP (2022) A GH115 α-glucuronidase structure reveals dimerization-mediated substrate binding and a proton wire potentially important for catalysis. Acta Crystallogr D Biol Crystallogr 78:658–668. 10.1107/S2059798322003527 10.1107/S2059798322003527 PMC9063842 35503213

[CR47] Wu H, Ho C, Ko T, Popat SD, Lin C, Wang AH-J (2010) Structural basis of α-fucosidase inhibition by iminocyclitols with *K*_i_ values in the micro- to picomolar range. Angew Chem Int Ed 49:337–340. 10.1002/anie.200905597 10.1002/anie.200905597 19967696

[CR48] Zehra S, Khambati I, Vierhout M, Mian MF, Buck R, Forsythe P (2018) Human milk oligosaccharides attenuate antigenantibody complex induced chemokine release from human intestinal epithelial cell lines. J Food Sci 83:499–508. 10.1111/1750-3841.14039 29377120 10.1111/1750-3841.14039

[CR49] Zeuner B, Meyer AS (2020) Enzymatic transfucosylation for synthesis of human milk oligosaccharides. Carbohydr Res 493:108029. 10.1016/j.carres.2020.108029 32445980 10.1016/j.carres.2020.108029

[CR50] Zeuner B, Teze D, Muschiol J, Meyer AS (2019) Synthesis of human milk oligosaccharides: protein engineering strategies for improved enzymatic transglycosylation. Molecules 24:2033. 10.3390/molecules24112033 31141914 10.3390/molecules24112033 PMC6600218

[CR51] Zhao C, Wu Y, Liu X, Liu B, Cao H, Yu H, Sarker SD, Nahar L, Xiao J (2017) Functional properties, structural studies and chemo-enzymatic synthesis of oligosaccharides. Trends Food Sci Technol 66:135–145. 10.1016/j.tifs.2017.06.008

[CR52] Zheng J, Xu H, Fang J, Zhang X (2022) Enzymatic and chemoenzymatic synthesis of human milk oligosaccharides and derivatives. Carbohydr Polym 291:119564.10.1016/j.carbpol.2022.119564 35698389 10.1016/j.carbpol.2022.119564

