# Supplementary material for: Calcium enhances hydrolytic and transfucosylation activities in α-L-fucosidase from Thermotoga maritima through catalytic loop stabilization: an MD simulation study
Source: Appl Microbiol Biotechnol. 2026 May 23;110:221. doi: 10.1007/s00253-026-13881-3 (PMC13375964; doi:10.1007/s00253-026-13881-3)
Supplement: Supplementary file 1 [file 253_2026_13881_MOESM1_ESM.pdf]

Calcium-induced stabilization of active-site loops enhances transfucosylation efficiency of α-L-fucosidase from *Thermotoga maritima*

Running title: Calcium enhances transfucosylation in FUC-*Tm*

Catalina Torres-Ochoa[a], Carlos Jiménez-Pérez[a], Salvador R. Tello-Solís[b], Sergio Alatorre-Santamaría[a], Francisco Guzmán-Rodríguez[a], Lorena Gómez-Ruiz[a], Alma Cruz-Guerrero[a*].

[a] Departamento de Biotecnología, Universidad Autónoma Metropolitana-Iztapalapa, Av. San Rafael Atlixco 186, Col. Vicentina, México 09340, Mexico.

[b] Departamento de Química, Universidad Autónoma Metropolitana-Iztapalapa, Av. San Rafael Atlixco 186, Col. Vicentina, México 09340, Mexico.

*Corresponding author: Alma Cruz-Guerrero

E-mail address: aec@xanum.uam.mx

ORCID: 0000-0002-9686-2267

Phone: +(52) (55) 5804-4720

Fax: +(52) (55) 5804-4712

Supporting Information

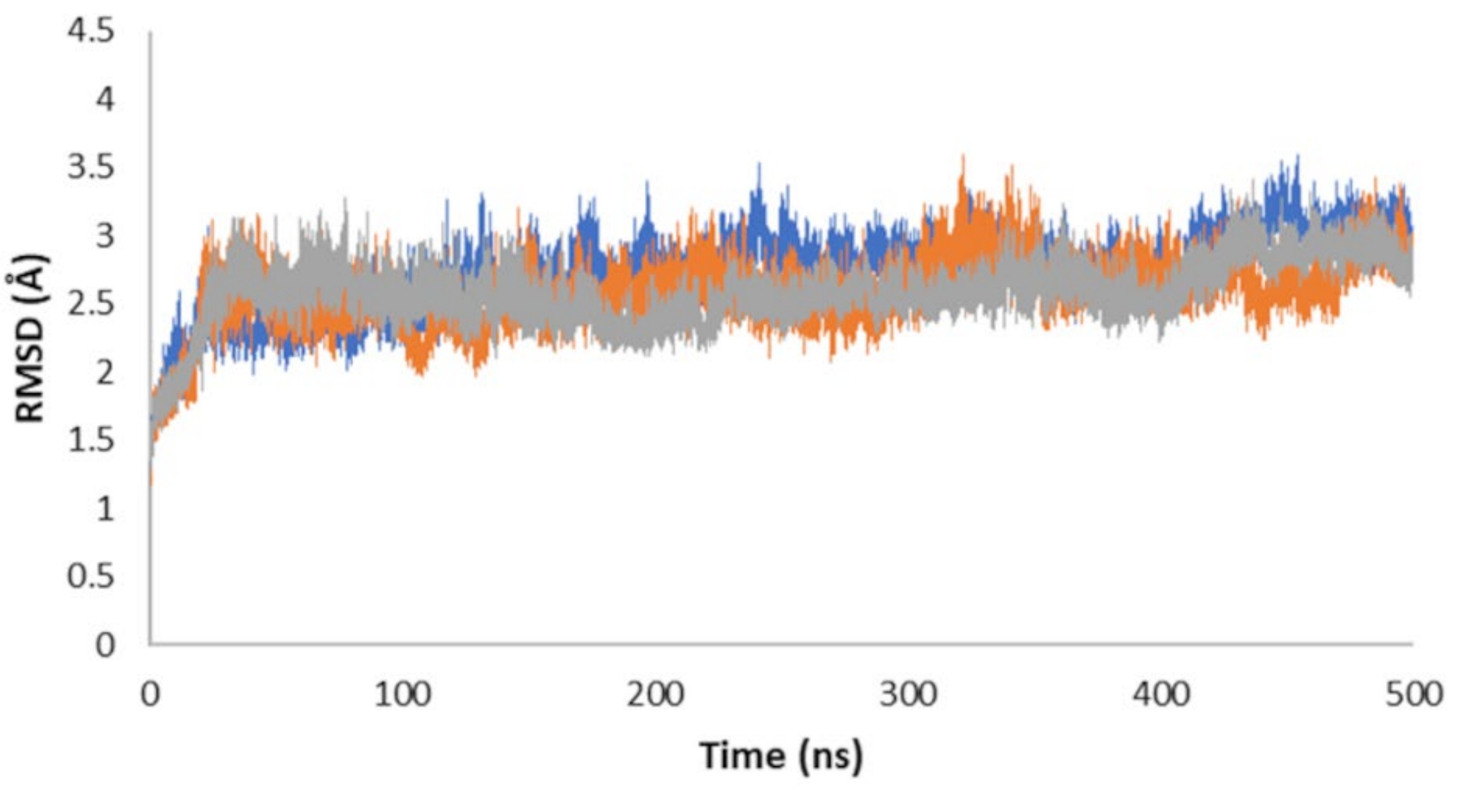

Fig. S1 RMSD of FUC-*Tm* in the system in the absence of Ca^{2+} over 500 ns of molecular dynamics simulations performed in triplicate.

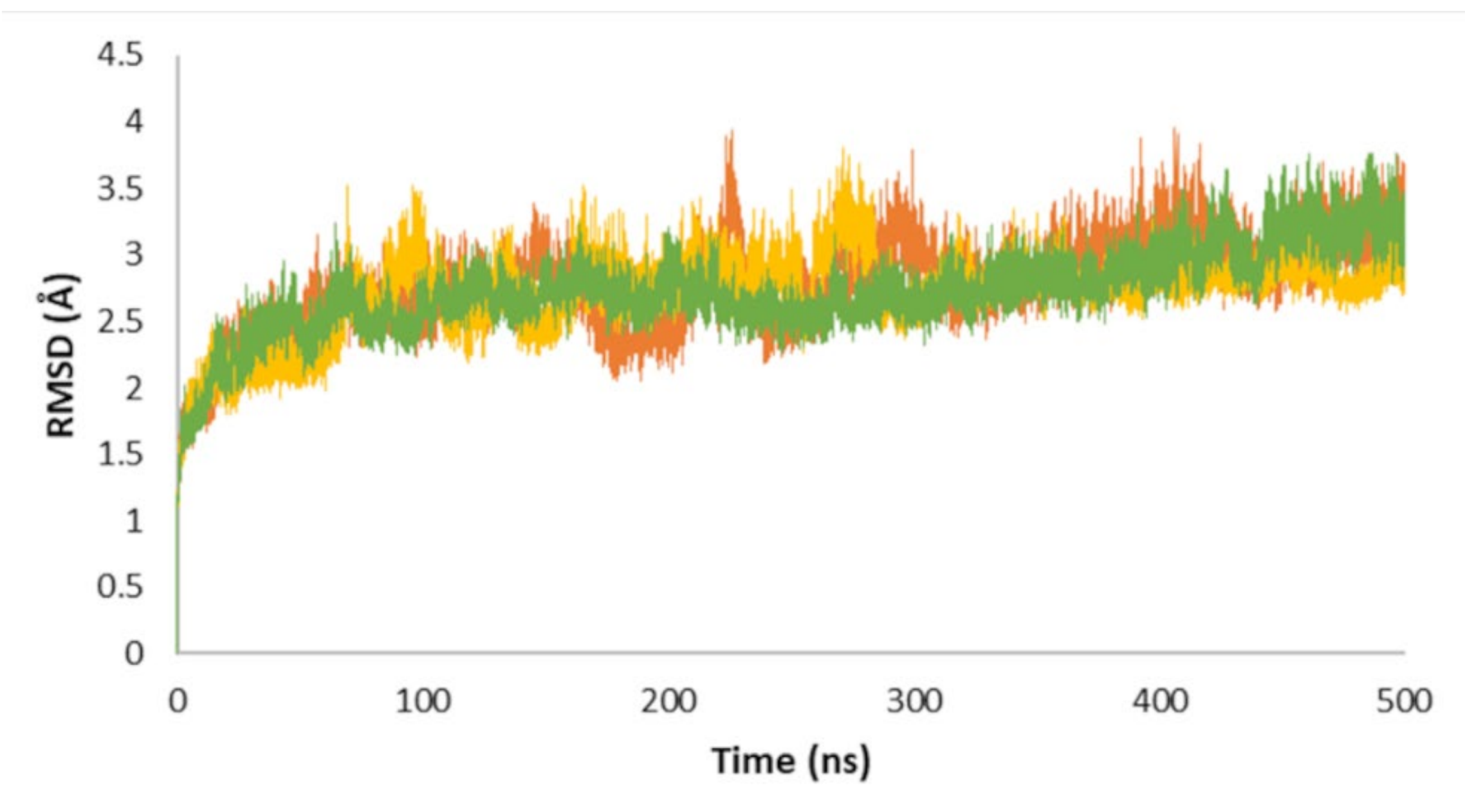

Fig. S2 RMSD of FUC-*Tm* in the system in the presence of Ca^{2+} over 500 ns of molecular dynamics simulations performed in triplicate.

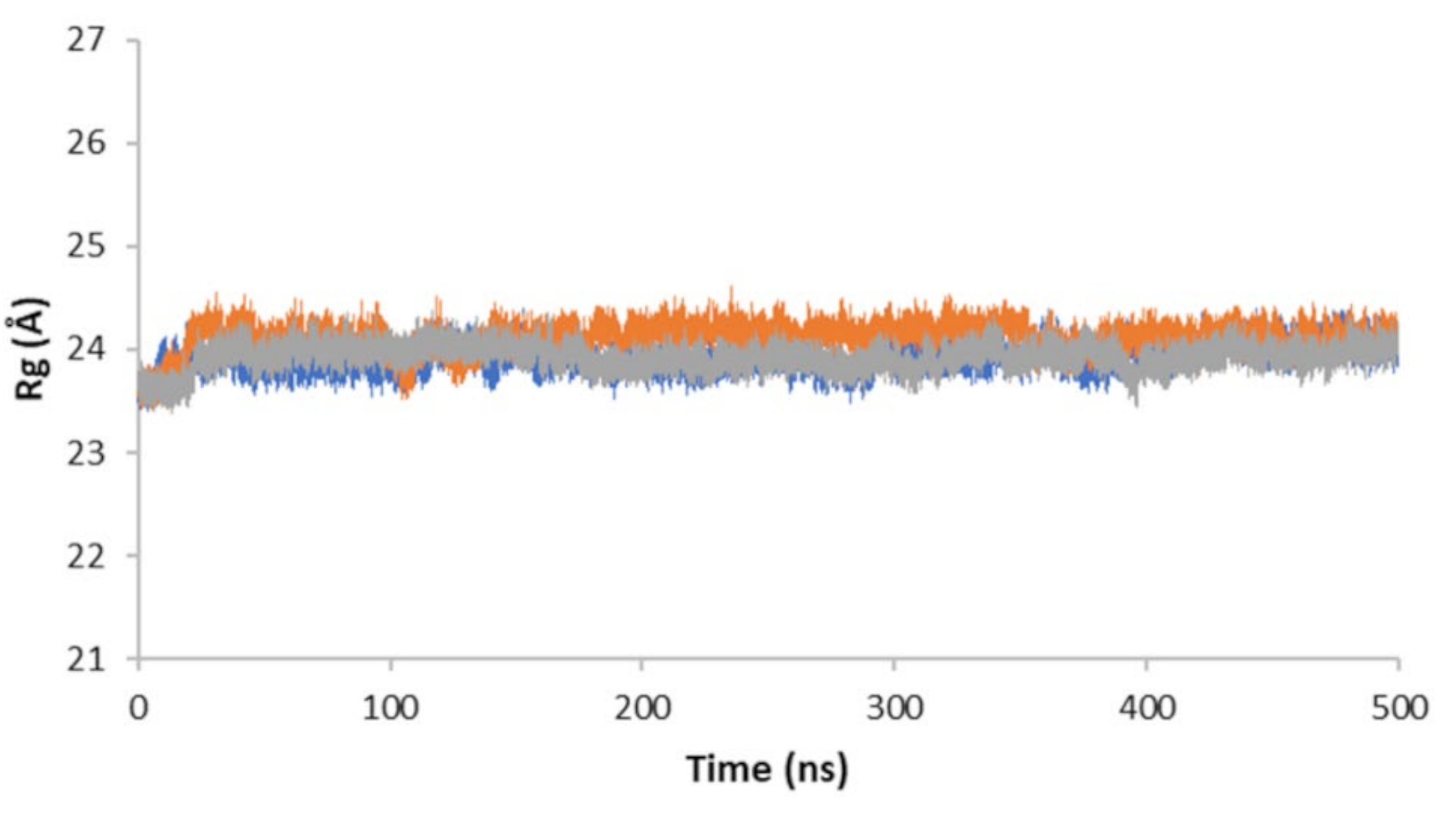

Fig. S3 Radius of gyration (Rg) of FUC-*Tm* in the system in the absence of Ca^{2+} over 500 ns of molecular dynamics simulations performed in triplicate.

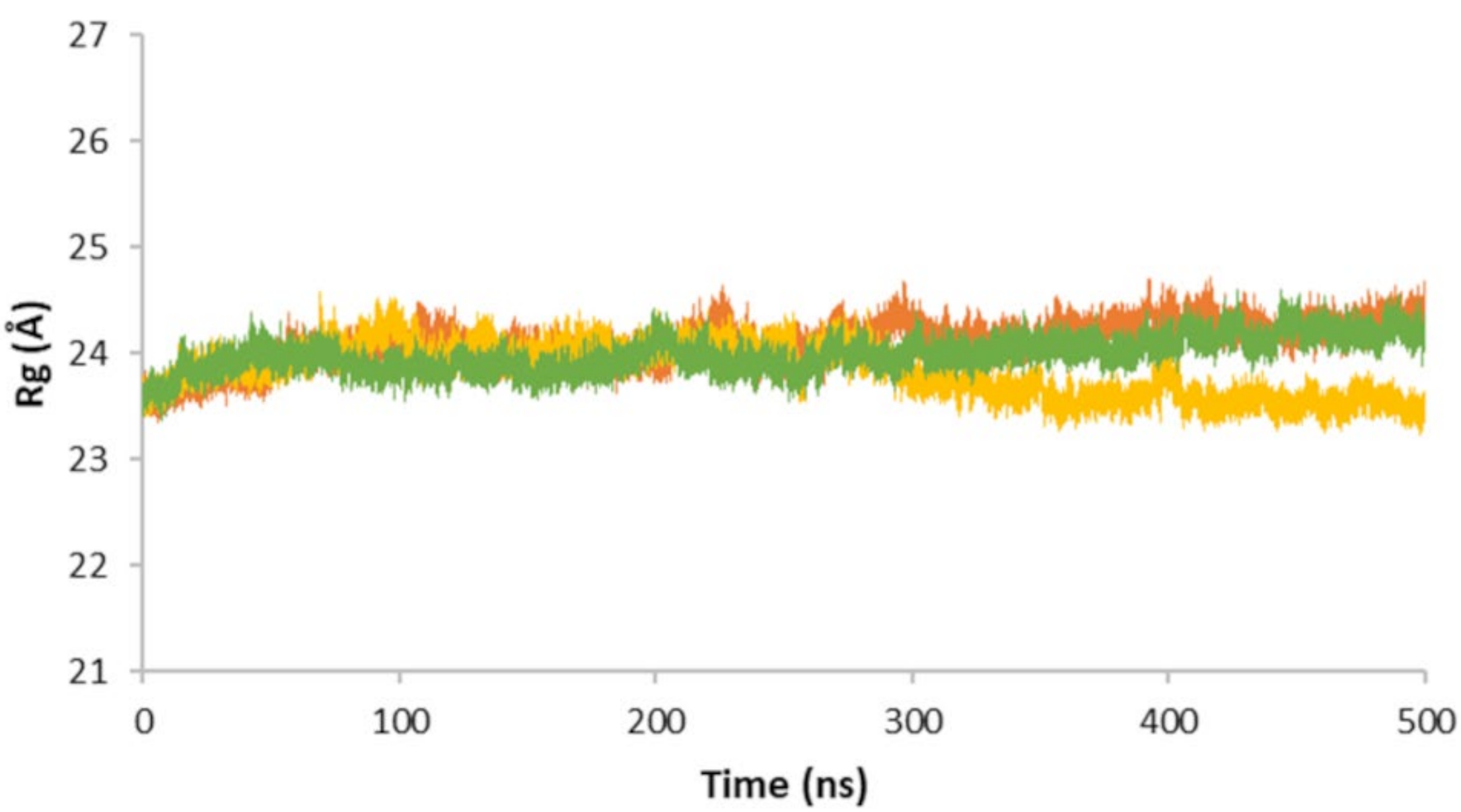

Fig. S4 Radius of gyration (Rg) of FUC-*Tm* in the system with Ca^{2+} (FUC-Tm–Ca^{2+}) over 500 ns of molecular dynamics simulations performed in triplicate.

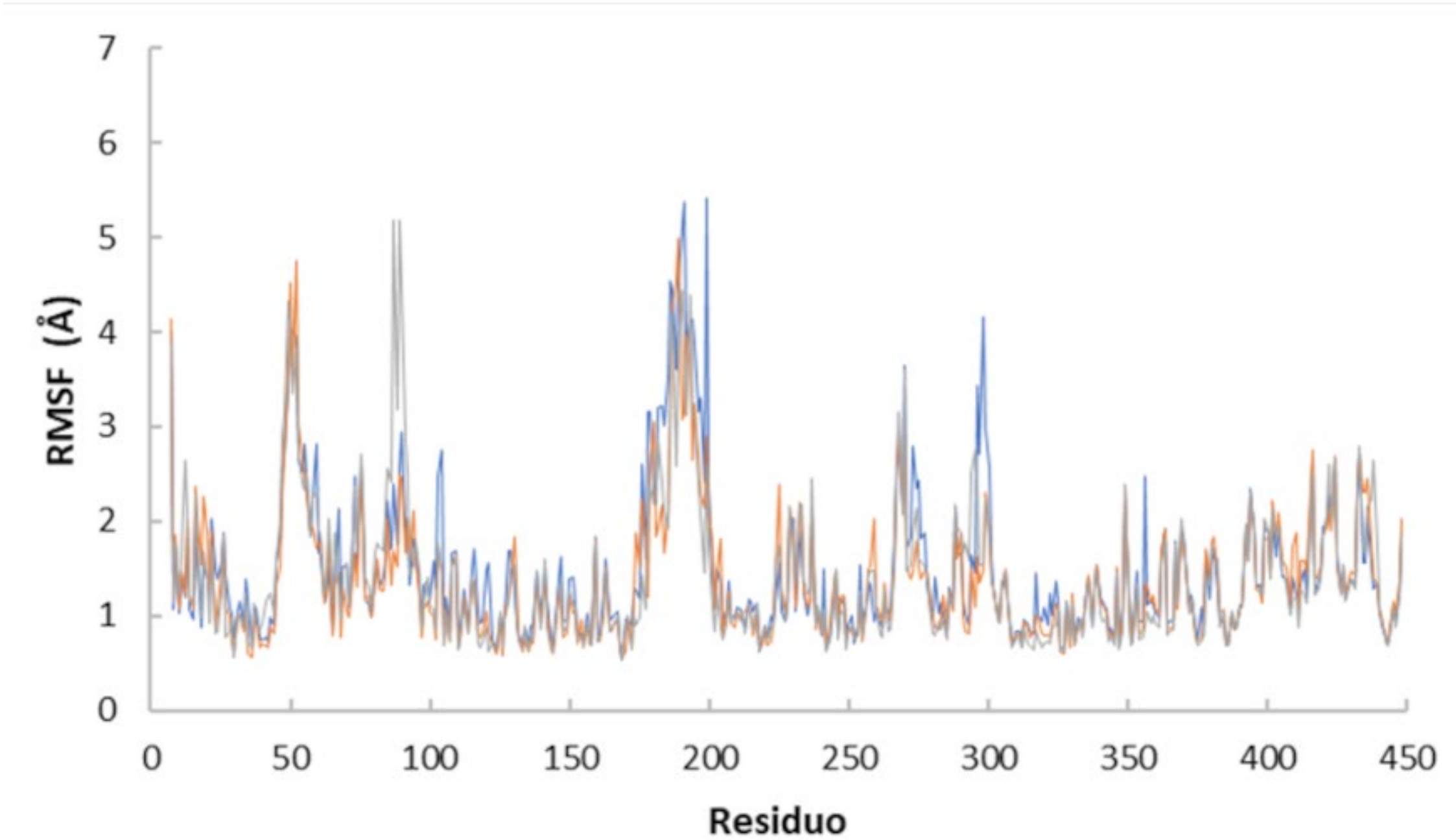

Fig. S5 RMSF of FUC-*Tm* in the system in the absence of Ca^{2+} over 500 ns of molecular dynamics simulations performed in triplicate.

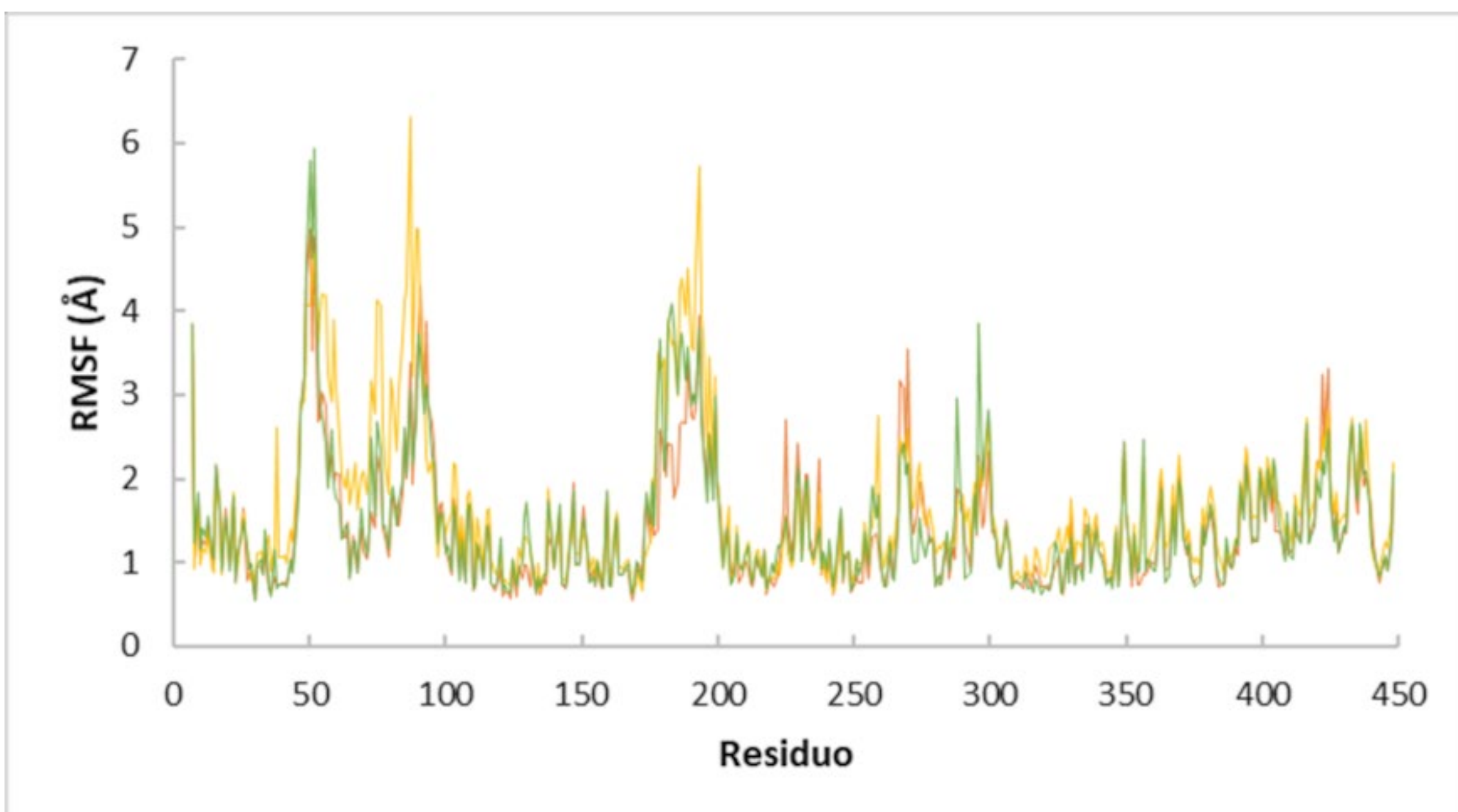

Fig. S6 RMSF of FUC-*Tm* in the system in the presence of Ca^{2+} over 500 ns of molecular dynamics simulations performed in triplicate.

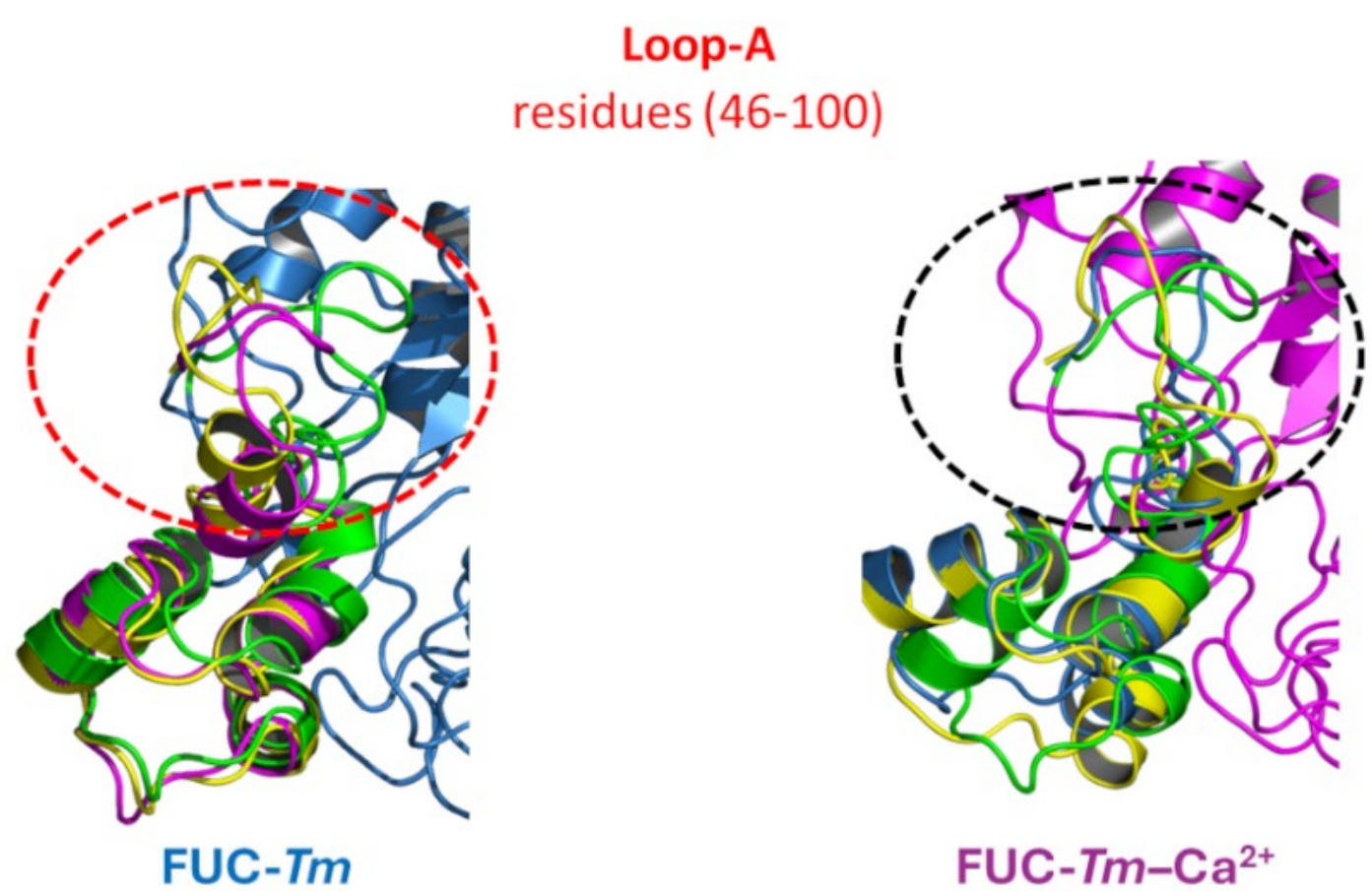

Without Ca^{2+}
Time (ns)
0.1
130.1
335.8

With Ca^{2+}
Time (ns)
0.1
130.1
335.8

Fig. S7 Time-resolved structural snapshots of Loop-A conformational dynamics. The dashed circles highlight the region of interest. Although increased RMSF values are observed in the presence of Ca^{2+}, the loop remains spatially confined near the catalytic cleft, consistent with RMSF analysis.

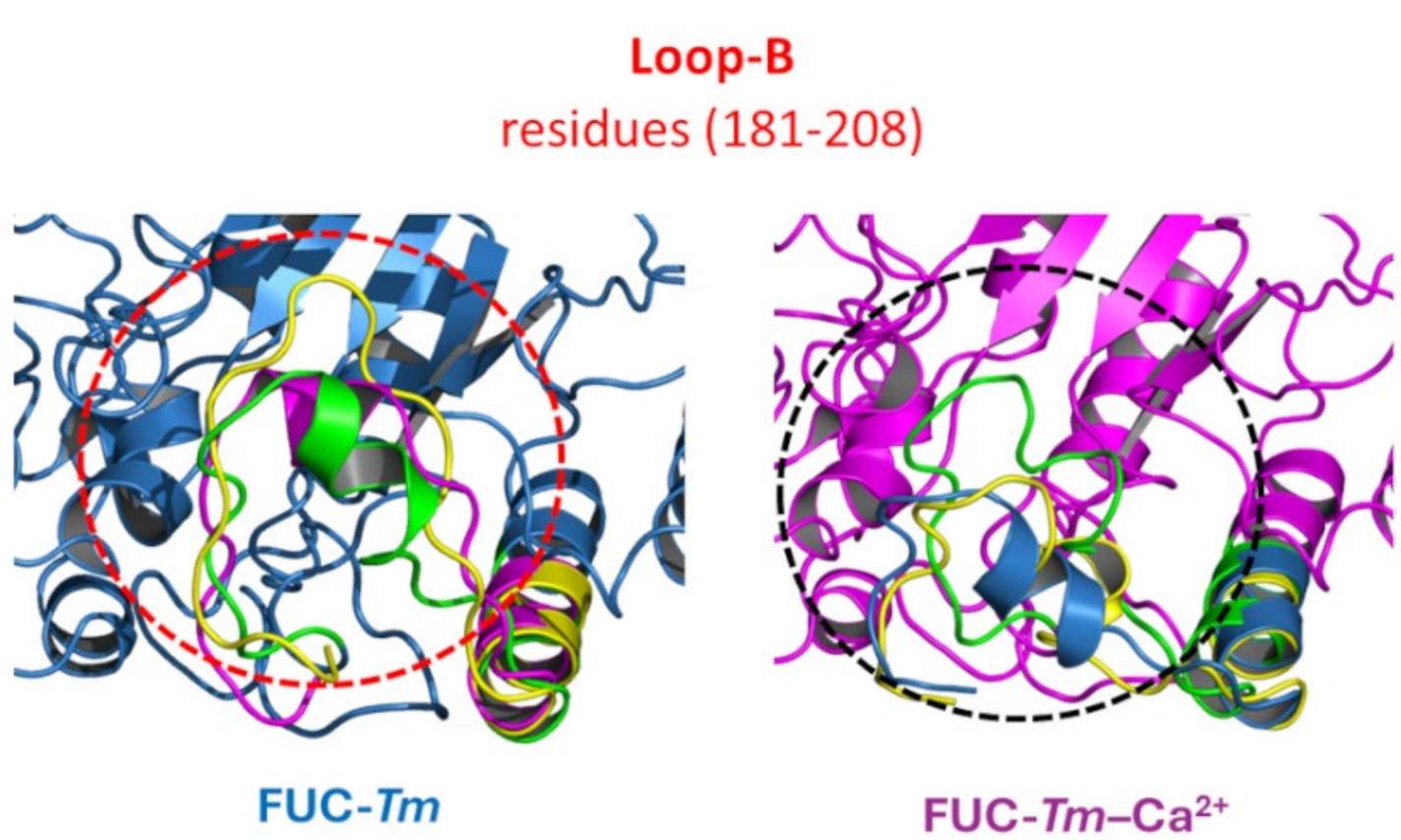

Without Ca^{2+}
Time (ns)
0.1
130.1
335.8

With Ca^{2+}
Time (ns)
0.1
130.1
335.8

Fig. S8 Time-resolved structural snapshots of Loop-B conformational dynamics. The dashed circles highlight the region of interest. Reduced conformational dispersion is observed in the Ca^{2+}-bound system, consistent with RMSF analysis.

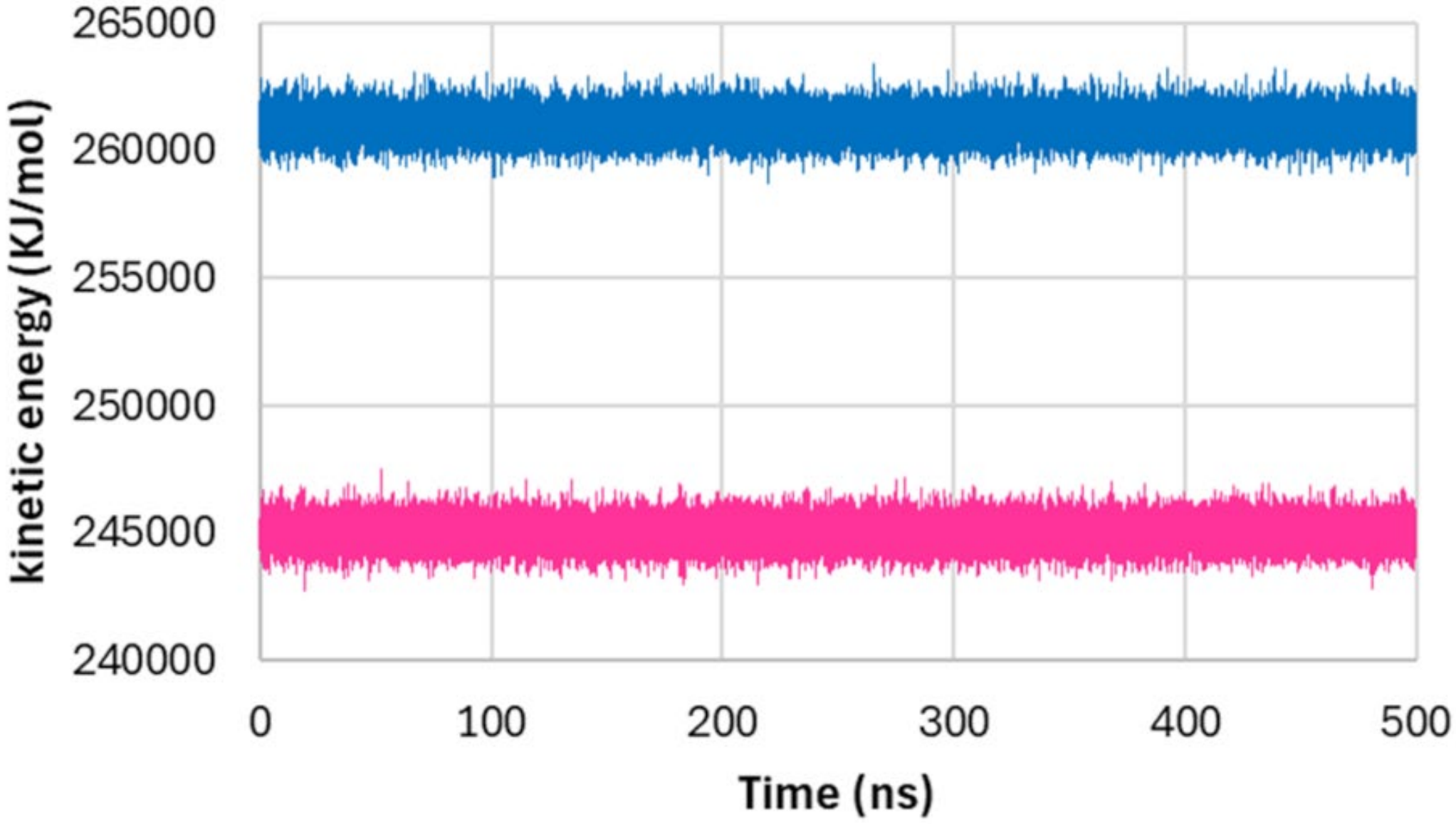

Fig. S9 Time evolution of kinetic energy during MD simulations in the absence and presence of Ca^{2+}. Kinetic energy profiles of FUC-*Tm* without Ca^{2+} (blue) and with Ca^{2+} (pink) over 500 ns of simulation. The Ca^{2+}-bound system exhibits consistently lower kinetic energy values, indicating reduced overall atomic mobility and supporting a more dynamically stable conformational state.

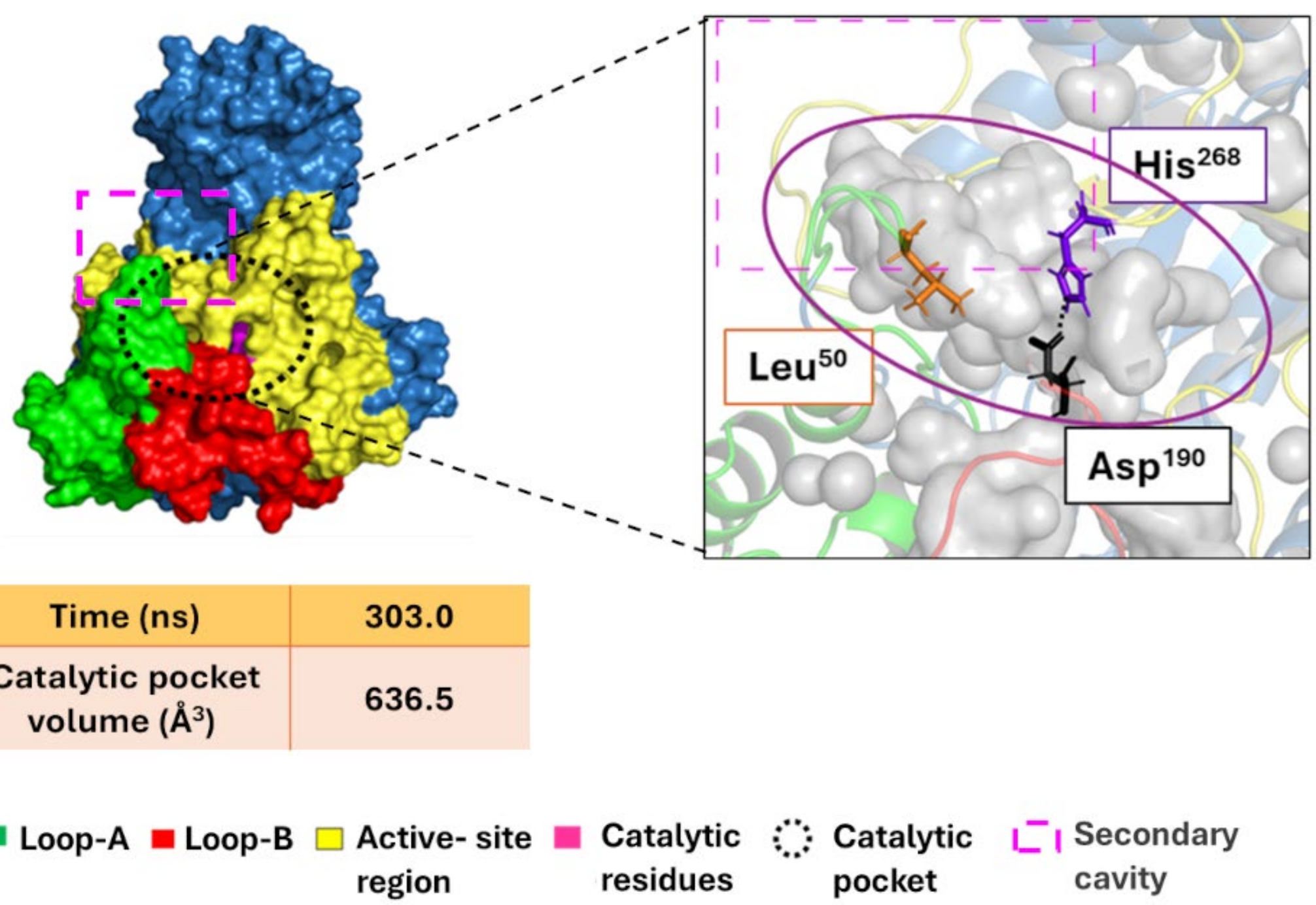

Time (ns)	303.0
Catalytic pocket volume (Å^3)	636.5

Fig. S10 Structural changes in the active-site cavity of FUC-*Tm* during MD simulation in the absence of Ca^{2+}. Snapshot at 303 ns. The active-site cavity is marked with circles, and dashed lines indicate residue–residue interactions.

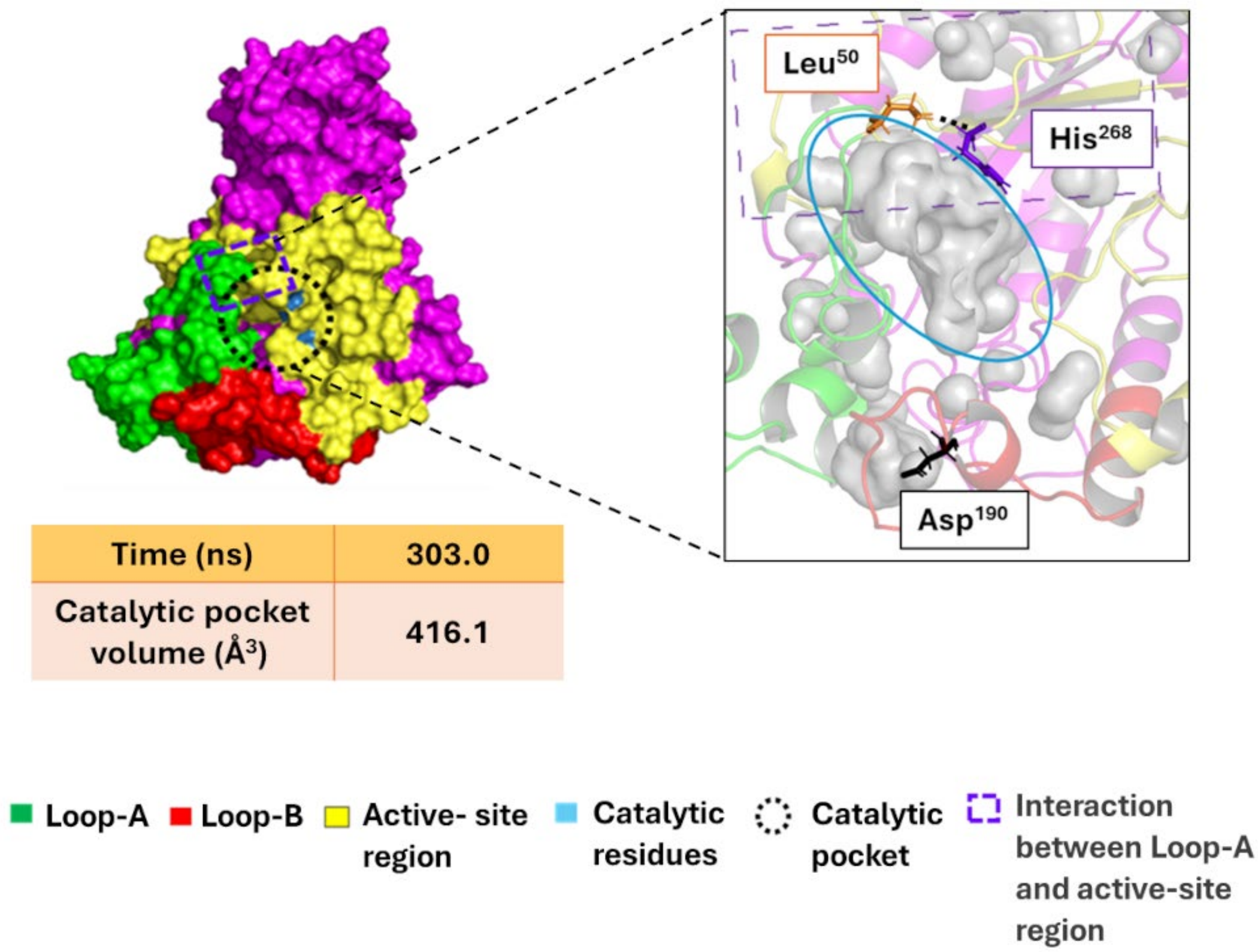

Fig. S11 Structural changes in the active-site cavity of FUC-*Tm* during MD simulation in the presence of Ca^{2+}. Snapshot at 303 ns. The active-site cavity is marked with circles, and dashed lines indicate residue–residue interactions.

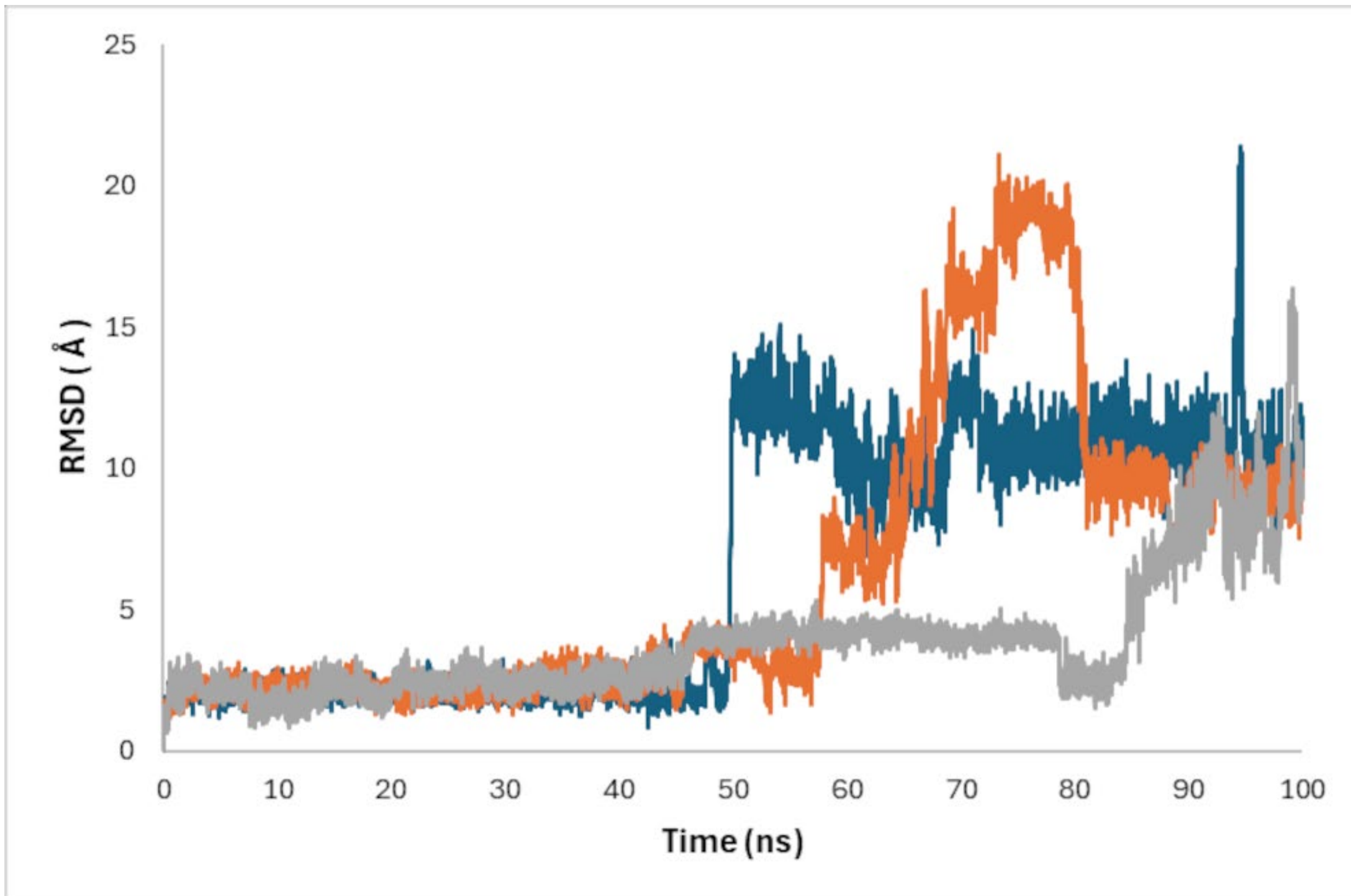

Fig. S12 RMSD of Complex in the system in the absence of Ca^{2+} over 100 ns of molecular dynamics simulations performed in triplicate.

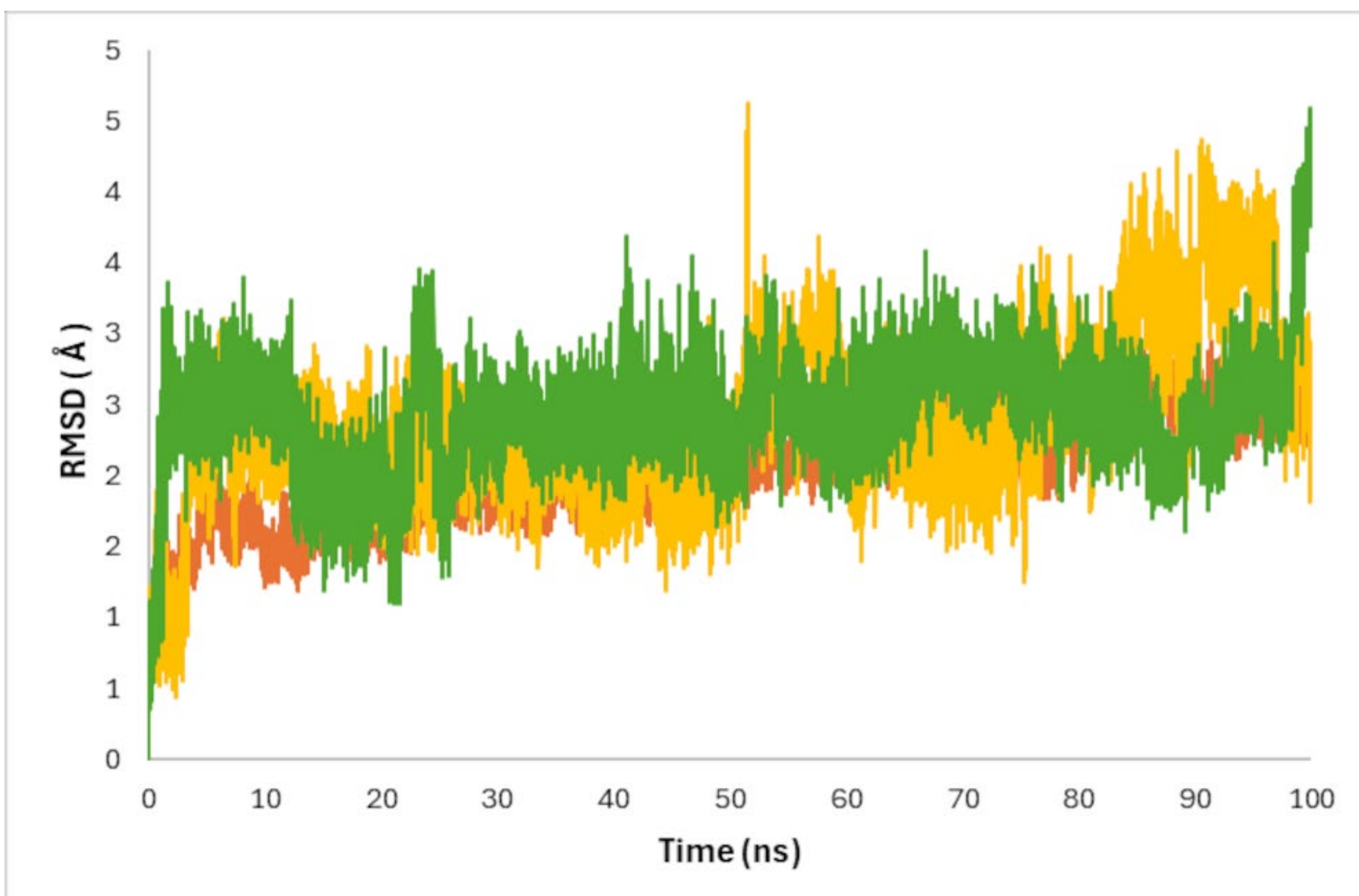

Fig. S13 RMSD of Complex in the system with Ca^{2+} over 100 ns of molecular dynamics simulations performed in triplicate.

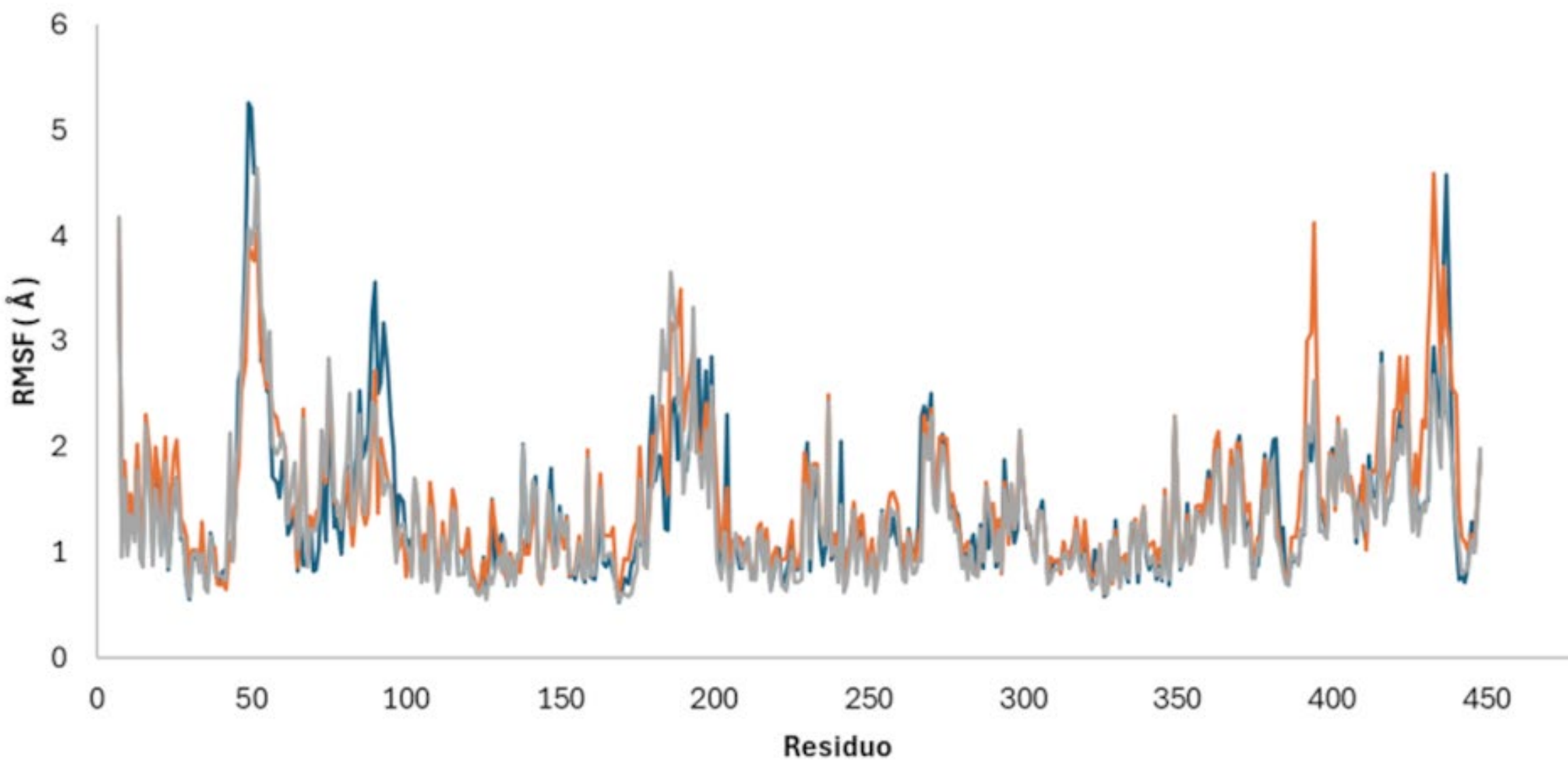

Fig. S14 RMSF of Complex in the system in the absence of Ca^{2+} over 100 ns of molecular dynamics simulations performed in triplicate.

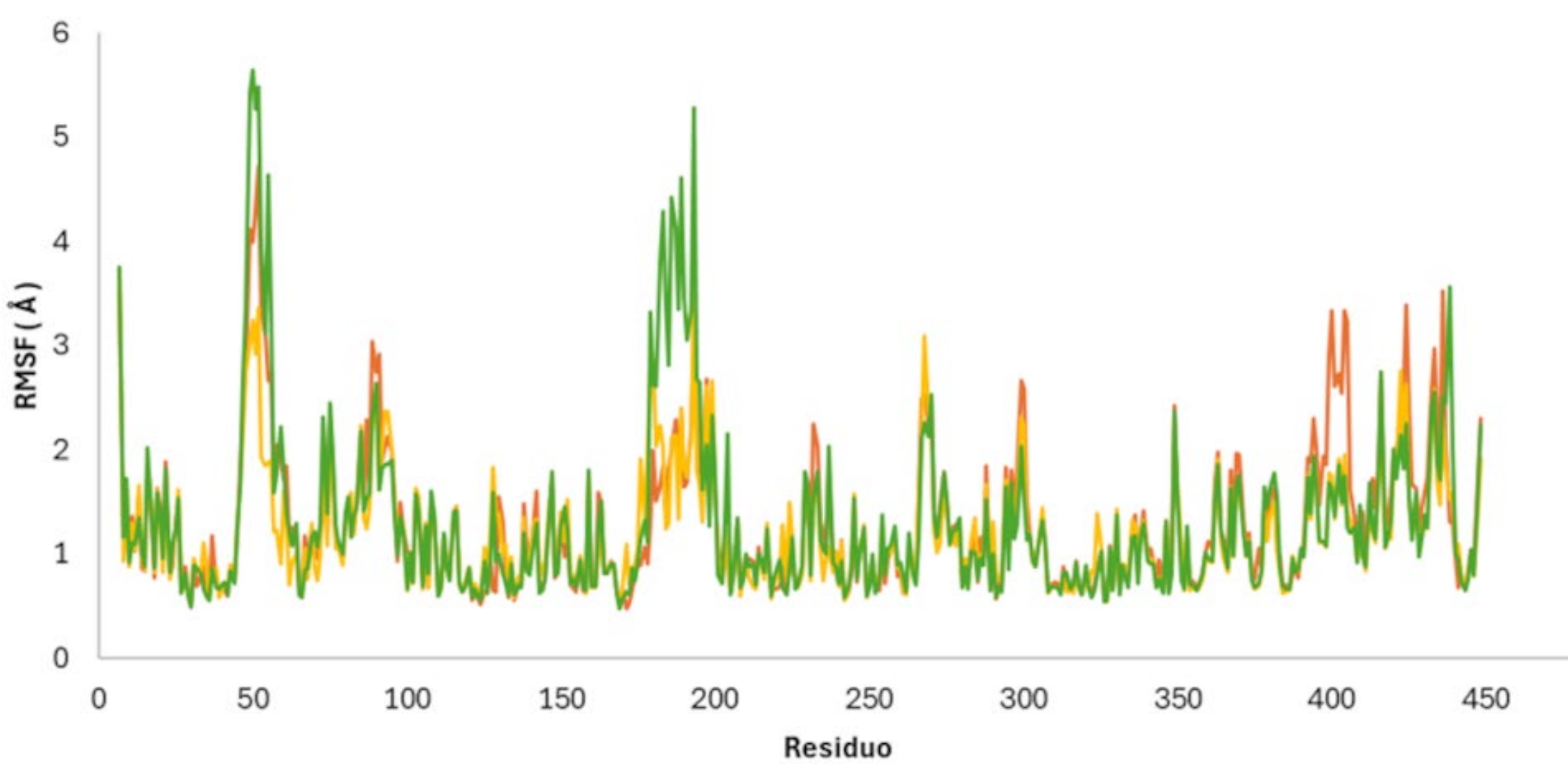

Fig. S15 RMSF of Complex in the system with Ca^{2+} over 100 ns of molecular dynamics simulations performed in triplicate.

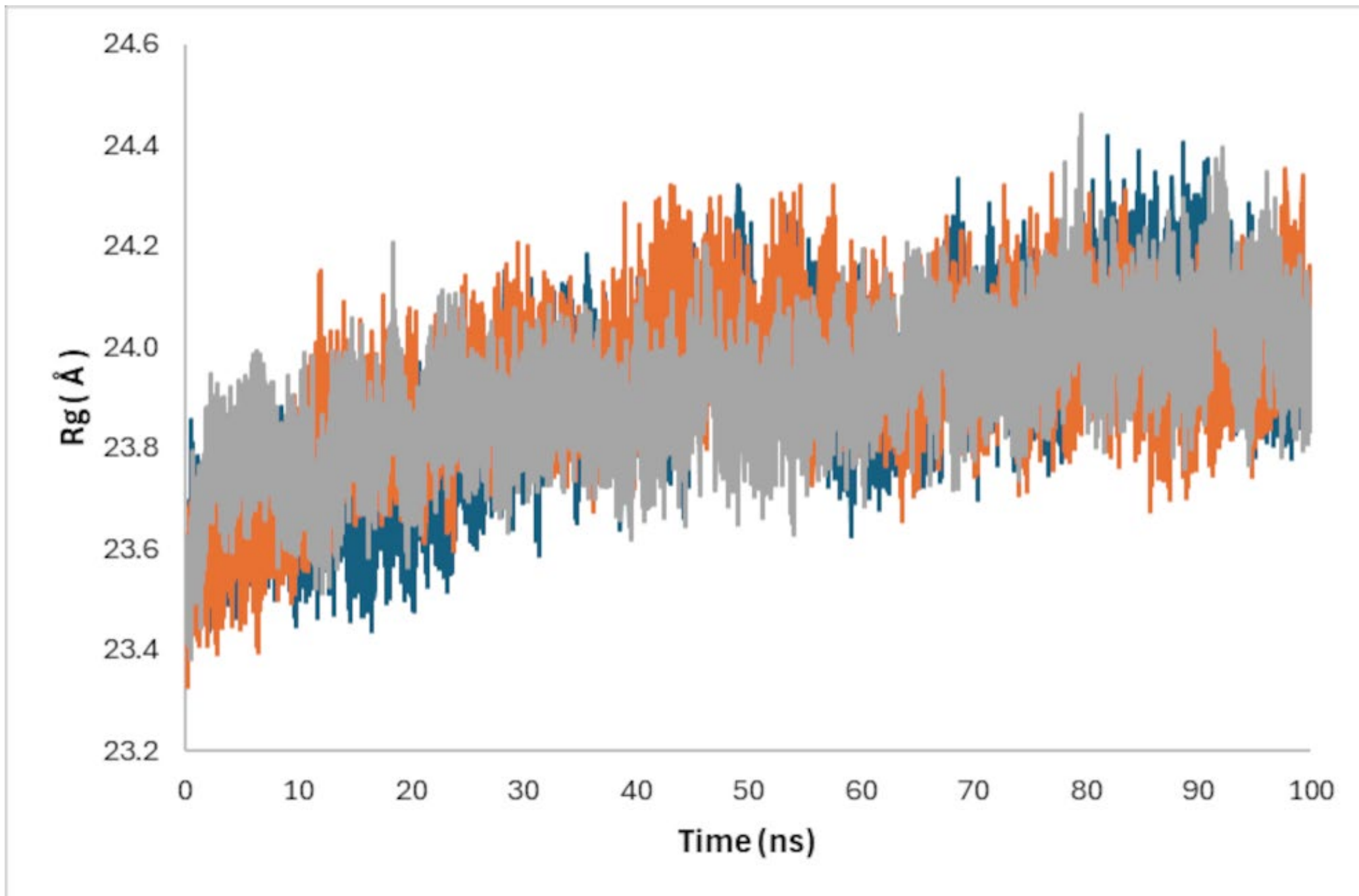

Fig. S16 Rg of Complex in the system in the absence of Ca^{2+} over 100 ns of molecular dynamics simulations performed in triplicate.

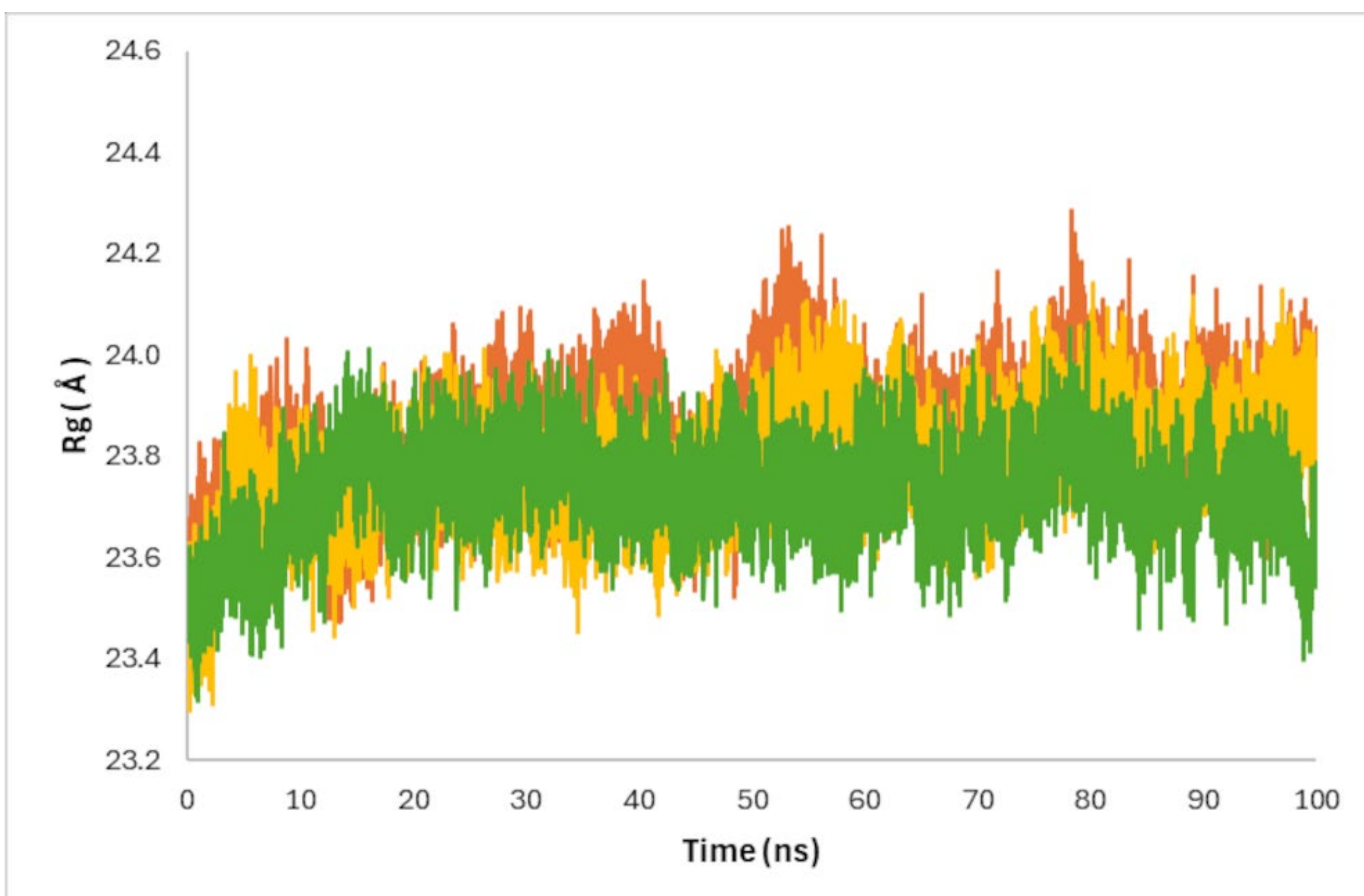

Fig. S17 Rg of Complex in the system with Ca^{2+} over 100 ns of molecular dynamics simulations performed in triplicate.
